# Supplementary material for: Mathematical modeling of the transmission of SARS-CoV-2—Evaluating the impact of isolation in São Paulo State (Brazil) and lockdown in Spain associated with protective measures on the epidemic of CoViD-19
Source: PLoS One. 2021 Jun 15;16(6):e0252271. doi: 10.1371/journal.pone.0252271 (PMC8205178; doi:10.1371/journal.pone.0252271)
Supplement: S1 Appendix — (PDF) [file pone.0252271.s001.pdf]

A Model formulation and analysis

We present a general mathematical model to describe the CoViD-19 epidemic and the calculation of the basic (R_0) and effective (R_{ef}) reproduction numbers. The general model aims to assess the effects of quarantine (isolation or lockdown) and further relaxation on the epidemic of CoViD-19.

A.1 The general model

In a community where SARS-CoV-2 (new coronavirus) is circulating, the risk of infection is more significant in elder than young persons, and elder persons are under an increased probability of being symptomatic and higher CoViD-19 induced mortality. Hence, the community is divided into two groups, composed of young (60 years old or less, denoted by subscript y) and elder (60 years old or more, denoted by subscript o) persons. This community's vital dynamic is described by the per-capita rates of birth (ϕ) and death (μ).

For each subpopulation j ($j = y, o$), all persons are divided into nine classes: susceptible S_j, susceptible persons who are isolated Q_j, exposed and incubating E_j, asymptomatic A_j, pre-symptomatic (or pre-diseased) persons before the onset of CoViD-19 D_{1j}, pre-diseased persons caught by test and then isolated Q_{1j}, symptomatic persons with severe CoViD-19 D_{2j}, mild CoViD-19 Q_{2j}, and mild CoViD-19 persons isolating themselves by educational campaign Q_{3j}. However, all young and elder persons in classes A_j, Q_{1j}, Q_{2j}, Q_{3j}, and D_{2j} enter into the same immune (recovered) class I (this is the 10th class, but common to both subpopulations).

The natural history of SARS-CoV-2 infection is the same for young ($j = y$) and elder ($j = o$) subpopulations. We assume that persons in the asymptomatic (A_j), pre-diseased (D_{1j}), and a fraction z_j of mild CoViD-19 (Q_{2j}) classes are transmitting the virus. Other infected classes (Q_{1j}, $(1 - z_j)\, Q_{2j}$ and D_{2j}) are under voluntary or forced isolation. Susceptible persons are infected at a rate $\lambda_j S_j$ (known as the mass action law [1]) and enter into class E_j, where λ_j is the per-capita incidence rate (or force of infection) defined by $\lambda_j = \lambda\,(\delta_{jy} + \psi\delta_{jo})$, with λ being

$$\lambda = \frac{1}{N}\frac{\varepsilon}{\omega}\left(\beta_{1y}A_y + \beta_{2y}D_{1y} + \beta_{3y}z_yQ_{2y} + \beta_{1o}A_o + \beta_{2o}D_{1o} + \beta_{3o}z_oQ_{2o}\right), \tag{A.1}$$

where δ_{ij} is the Kronecker delta, with $\delta_{ij} = 1$ if $i = j$, and 0, if $i \neq j$; and β_{1j}, β_{2j} and β_{3j} are the transmission rates, that is, the rates at which a virus encounters a susceptible people and infects him/her. The parameters $\varepsilon \leq 1$ and $\omega \geq 1$ diminish the transmission rates. The protection factor ε decreases the transmission of infection by individual (face mask, hygiene, etc.) and collective (social distancing) protective measures, while the reduction factor ω decreases the transmission by the contact being restricted in the population in isolation (household and neighborhood contacts).

Susceptible persons are infected at a rate λ_j and enter into class E_j. After an average period $1/\sigma_j$ in class E_j, where σ_j is the incubation rate, exposed persons enter into the asymptomatic class A_j (with probability p_j) or pre-diseased class D_{1j} (with probability $1 - p_j$). After an average period $1/\gamma_j$ in class A_j, where γ_j is the recovery rate of asymptomatic persons, asymptomatic persons acquire immunity and enter into immune (recovered) class I. Possibly asymptomatic persons can manifest symptoms at the end of this period, and a fraction $1 - \chi_j$ enters into mild CoViD-19 class Q_{2j}. Another route of exit from class A_j is being caught by a test at a rate η_j and enter into class I (we assume that this person indeed adopts isolation, which is the reason to enter into class I at a rate of testing). For symptomatic persons, after an average period $1/\gamma_{1j}$ in class D_{1j}, where γ_{1j} is the infection rate of pre-diseased persons, pre-diseased persons enter into severe CoViD-19 class D_{2j} (with probability $1 - m_j$) or class Q_{2j} (with probability m_j), or they are caught by test at a rate η_{1j} and enter into class Q_{1j}. Persons in class D_2 acquire immunity after period $1/\gamma_{2j}$, where γ_{2j} is the recovery rate of severe CoViD-19, and enter into class I or die under the disease-induced (additional) mortality rate α_j. Another route of exiting class D_{2j} is by treatment, described by the treatment rate θ_j. Class Q_{1j} is composed of mild and severe CoViD-19 persons who came from class D_{1j} caught by test; hence they enter into class D_{2j} (with the rate $(1 - m_j)\gamma_{1j}$) or class I (with the rate $m_j\gamma_{1j} + \gamma_{2j}$, assuming adherence to isolation). Persons in class Q_{2j} acquire immunity after period $1/\gamma_{3j}$, where γ_{3j} is the recovery rate of mild CoViD-19, and enter into immune class I. Another route of exit from class Q_{2j} are being caught by a test at a rate η_{2j} and enter into class I (assumption of adherence to isolation), or enter to class Q_{3y} convinced by an education campaign at a rate ϖ_j, which is temporary; hence ξ_j is the rate of abandonment of protective measures [2].

In the model, we consider pulse isolation and intermittent (series of pulses) release of persons. We assume that there is a unique pulse in isolation at time $t = \tau_j^{is}$, described by $k_j S_j \delta\left(t - \tau_j^{is}\right)$, but there are m intermittent releases described by $\sum_{i=1}^{m} l_{ij} Q_j \delta\left(t - t_i\right)$, where $t_i = \tau_j^{is} + \sum_{w=1}^{i} \tau_{wj}$, $j = y, o$, and $\delta\left(x\right)$ is the Dirac delta function, that is, $\delta\left(x\right) = \infty$, if $x = 0$, otherwise, $\delta\left(x\right) = 0$, with $\int_0^\infty \delta\left(x\right) dx = 1$. The fraction of persons in isolation is k_j, and l_{ij}, $i = 1, 2, \cdots, m$, is the fraction of i-th release of isolated persons, with τ_{wj} being the period between successive releases.

Fig A.1 shows the flowchart of the SARS-CoV-2 transmission model.

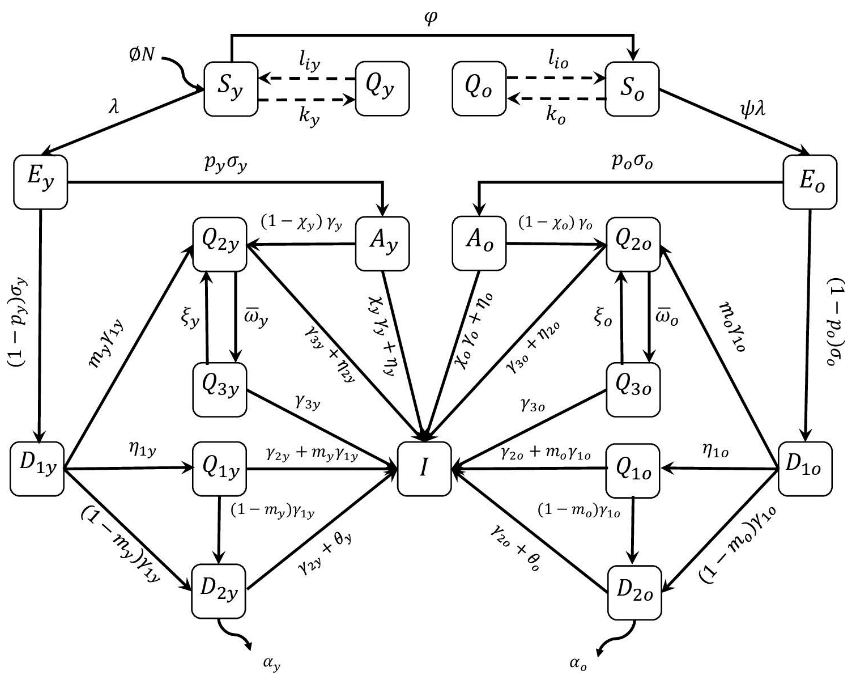

Fig A.1. The flowchart of new coronavirus transmission model with variables and parameters.

Based on the above descriptions summarized in Fig A.1, the new coronavirus transmission model is described by the system of ordinary differential equations, with

$j = y, o$. Equations for susceptible and isolated persons are

$$\begin{cases} \frac{d}{dt}S_y &= \phi N - (\varphi + \mu) S_y - \lambda S_y - k_y S_y \delta\left(t - \tau_y^{is}\right) + \sum_{i=1}^{m} l_{iy} Q_y \delta\left(t - \tau_j^{is} - \sum_{w=1}^{i} \tau_{wy}\right) \\ \frac{d}{dt}S_o &= \varphi S_y - \mu S_o - \lambda \psi S_o - k_o S_o \delta\left(t - \tau_o^{is}\right) + \sum_{i=1}^{m} l_{io} Q_o \delta\left(t - \tau_j^{is} - \sum_{w=1}^{i} \tau_{wo}\right) \\ \frac{d}{dt}Q_j &= k_j S_j \delta\left(t - \tau_j^{is}\right) - \mu Q_j - \sum_{i=1}^{m} l_{ij} Q_j \delta\left(t - \tau_j^{is} - \sum_{w=1}^{i} \tau_{wj}\right), \end{cases} \tag{A.2}$$

for incubating and infectious persons,

$$\begin{cases} \frac{d}{dt}E_j &= \lambda\left(\delta_{jy} + \psi\delta_{jo}\right) S_j - (\sigma_j + \mu) E_j \\ \frac{d}{dt}A_j &= p_j \sigma_j E_j - (\gamma_j + \eta_j + \mu) A_j \\ \frac{d}{dt}D_{1j} &= (1 - p_j) \sigma_j E_j - (\gamma_{1j} + \eta_{1j} + \mu) D_{1j} \\ \frac{d}{dt}Q_{2j} &= (1 - \chi_j) \gamma_j A_j + m_j \gamma_{1j} D_{1j} + \xi_j Q_{3j} - (\gamma_{3j} + \eta_{2j} + \varpi_j + \mu) Q_{2j} \\ \frac{d}{dt}Q_{3j} &= \varpi_j Q_{2j} - (\gamma_{3j} + \xi_j + \mu) Q_{3j} \\ \frac{d}{dt}Q_{1j} &= \eta_{1j} D_{1j} - (\gamma_{2j} + \gamma_{1j} + \mu) Q_{1j} \\ \frac{d}{dt}D_{2j} &= (1 - m_j) \gamma_{1j} (D_{1j} + Q_{1j}) - (\gamma_{2j} + \theta_j + \mu + \alpha_j) D_{2j}, \end{cases} \tag{A.3}$$

and for immune (recovered) persons,

$$\begin{aligned} \frac{d}{dt}I = \; & (\chi_y \gamma_y + \eta_y) A_y + (\gamma_{2y} + m_y \gamma_{1y}) Q_{1y} + (\gamma_{3y} + \eta_{2y}) Q_{2y} + \gamma_{3y} Q_{3y} + \\ & (\gamma_{2y} + \theta_y) D_{2y} + (\chi_o \gamma_o + \eta_o) A_o + (\gamma_{2o} + m_o \gamma_{1o}) Q_{1o} + (\gamma_{3o} + \eta_{2o}) Q_{2o} + \\ & \gamma_{3o} Q_{3o} + (\gamma_{2o} + \theta_o) D_{2o} - \mu I, \end{aligned} \tag{A.4}$$

where $N_j = S_j + Q_j + E_j + A_j + D_{1j} + Q_{1j} + Q_{2j} + Q_{3j} + D_{2j}$, and $N = N_y + N_o + I$ obeys

$$\frac{d}{dt}N = (\phi - \mu) N - \alpha_y D_{2y} - \alpha_o D_{2o}, \tag{A.5}$$

with the initial number of population at $t = 0$ being $N(0) = N_0 = N_{0y} + N_{0o}$, where N_{0y} and N_{0o} are the size of young and elder subpopulations at $t = 0$. If $\phi = \mu + (\alpha_y D_{2y} + \alpha_o D_{2o}) / N$, the total size of the population is constant.

The model variables are those given in Table 1 in the main text plus Q_{1j} (Pre-diseased persons caught by a test) and Q_{3j} (Mild CoViD-19 persons adhered to isolation). The model parameters are those given in Table 2 in the main text plus summarized in Table A.1.

A.2 The steady-state analysis – R_0 and R_{ef}

The basic reproduction number R_0 is obtained by the analysis of the trivial equilibrium point in the steady-state. However, the non-autonomous and varying population system of Eqs (A.2), (A.3), and (A.4) does not have a steady-state. Observe that the population in isolation is maintained for a while; hence, disregarding this period of isolation, the system of equations is autonomous (we let $k_j = l_{ij} = 0$, $j = y, o$), but the population N varies. For this reason, considering the fractions of individuals in each compartment defined by

$$x_j = \frac{X_j}{N}, \quad \text{where} \quad X = S_j, Q_j, E_j, A_j, Q_{1_j}, D_{1j}, Q_{2j}, Q_{3j}, D_{2j}, I,$$

Table A.1. Summary of the general model parameters ($j = y, o$).

Symbol	Meaning
$\eta_y\,(\eta_o)$	Testing rate among asymptomatic persons
$\eta_{1y}\,(\eta_{1o})$	Testing rate among pre-diseased persons
$\eta_{2y}\,(\eta_{2o})$	Testing rate among mild CoViD-19 persons
$\varpi_y\,(\varpi_o)$	Adherence to protection behavior rate
$\xi_y\,(\xi_o)$	Loss of protection behavior rate
$l_{1y}\,(l_{1o})$	Proportion released at first time t_1
$\tau_{1y}(\tau_{1o})$	Time of the first (i-th) releasing
$\theta_y\,(\theta_o)$	Treatment rate
ψ	Scaling factor of transmission among elder persons

and using Eq (A.5) for N, we obtain

$$\frac{d}{dt}x_j \equiv \frac{d}{dt}\frac{X_j}{N} = \frac{1}{N}\frac{d}{dt}X_j - x_j\frac{1}{N}\frac{d}{dt}N = \frac{1}{N}\frac{d}{dt}X_j - x\left(\phi-\mu\right) + x_j\left(\alpha_y d_{2y} + \alpha_o d_{2o}\right).$$

Hence, the system of Eqs (A.2), (A.3), and (A.4) in terms of fractions becomes, for susceptible and isolated persons,

$$\left\{\begin{array}{lcl} \frac{d}{dt}s_y & = & \phi - \left(\varphi+\phi\right)s_y - \lambda s_y + s_y\left(\alpha_y d_{2y}+\alpha_o d_{2o}\right) \\ \frac{d}{dt}s_o & = & \varphi s_y - \phi s_o - \lambda\psi s_o + s_o\left(\alpha_y d_{2y}+\alpha_o d_{2o}\right), \\ \frac{d}{dt}q_j & = & -\phi q_j + q_j\left(\alpha_y d_{2y}+\alpha_o d_{2o}\right), \end{array}\right. \tag{A.6}$$

for infected persons,

$$\left\{\begin{array}{lcl} \frac{d}{dt}e_j & = & \lambda\left(\delta_{jy}+\psi\delta_{jo}\right)s_j - \left(\sigma_j+\phi\right)e_j + e_j\left(\alpha_y d_{2y}+\alpha_o d_{2o}\right) \\ \frac{d}{dt}a_j & = & p_j\sigma_j e_j - \left(\gamma_j+\eta_j+\phi\right)a_j + a_j\left(\alpha_y d_{2y}+\alpha_o d_{2o}\right) \\ \frac{d}{dt}d_{1j} & = & \left(1-p_j\right)\sigma_j e_j - \left(\gamma_{1j}+\eta_{1j}+\phi\right)d_{1j} + d_{1j}\left(\alpha_y d_{2y}+\alpha_o d_{2o}\right) \\ \frac{d}{dt}q_{2j} & = & \left(1-\chi_j\right)\gamma_j a_j + m_j\gamma_{1j}d_{1j} + \xi_j q_{3j} - \left(\gamma_{3j}+\eta_{2j}+\varpi_j+\phi\right)q_{2j} + \\ & & +q_{2j}\left(\alpha_y d_{2y}+\alpha_o d_{2o}\right) \\ \frac{d}{dt}q_{3j} & = & \varpi_j q_{2j} - \left(\gamma_{3j}+\xi_j+\phi\right)q_{3j} + q_{3j}\left(\alpha_y d_{2y}+\alpha_o d_{2o}\right) \\ \frac{d}{dt}q_{1j} & = & \eta_{1j}d_{1j} - \left(\gamma_{2j}+\gamma_{1j}+\phi\right)q_{1j} + q_{1j}\left(\alpha_y d_{2y}+\alpha_o d_{2o}\right) \\ \frac{d}{dt}d_{2j} & = & \left(1-m_j\right)\gamma_{1j}\left(d_{1j}+q_{1j}\right) - \left(\gamma_{2j}+\theta_j+\phi+\alpha_j\right)d_{2j} + d_{2j}\left(\alpha_y d_{2y}+\alpha_o d_{2o}\right), \end{array}\right. \tag{A.7}$$

and for immune (recovered) persons,

$$\begin{array}{lcl} \frac{d}{dt}i & = & \left(\chi_y\gamma_y+\eta_y\right)a_y + \left(\gamma_{2y}+m_y\gamma_{1y}\right)q_{1y} + \left(\gamma_{3y}+\eta_{2y}\right)q_{2y} + \gamma_{3y}q_{3y} + \left(\gamma_{2y}+\theta_y\right)d_{2y} + \\ & & \left(\chi_o\gamma_o+\eta_o\right)a_o + \left(\gamma_{2o}+m_o\gamma_{1o}\right)q_{1o} + \left(\gamma_{3o}+\eta_{2o}\right)q_{2o} + \gamma_{3o}q_{3o} + \left(\gamma_{2o}+\theta_o\right)d_{2o} - \\ & & \phi i + i\left(\alpha_y d_{2y}+\alpha_o d_{2o}\right), \end{array} \tag{A.8}$$

where λ is the force of infection given by Eq (A.1) re-written as

$$\lambda = \frac{\varepsilon}{\omega}\left(\beta_{1y}a_y + \beta_{2y}d_{1y} + \beta_{3y}z_y q_{2y} + \beta_{1o}a_o + \beta_{2o}d_{1o} + \beta_{3o}z_o q_{2o}\right),$$

and

$$\sum_{j=y,o}\left(s_j + q_j + e_j + a_j + q_{1j} + d_{1j} + q_{2j} + q_{3j} + d_{2j}\right) + i = 1.$$

This new system of equation attains a steady-state, that is, the number of persons in all classes varies with time however, their fractions approach the steady-state (the sum of derivatives of all classes is zero).

A.2.1 The basic reproduction number R_0 – The trivial equilibrium point and its stability

The trivial (disease-free) equilibrium point P^0 of the new system of Eqs (A.6), (A.7) and (A.8) is given by

$$P^0 = \left(s_j^0, q_j^0 = 0, e_j^0 = 0, a_j^0 = 0, q_{1j}^0 = 0, d_{1j}^0 = 0, q_{2j}^0 = 0, q_{3j}^0 = 0, d_{2j}^0 = 0, i^0 = 0\right),$$

for $j = y$ and o, where

$$\begin{cases} s_y^0 = \dfrac{\phi}{\phi+\varphi} \\ s_o^0 = \dfrac{\varphi}{\phi+\varphi}, \end{cases} \tag{A.9}$$

with $s_y^0 + s_o^0 = 1$.

Let us assess the stability of P^0 by applying the next generation matrix theory considering the vector of variables $x = (e_y, a_y, d_{1y}, q_{2y}, e_o, a_o, d_{1o}, q_{2o})$ [3]. We apply method proposed in [4] and proved in [5]. Due to the control mechanisms, we obtain the reduced reproduction number R_c.

To obtain the reduced reproduction number, the diagonal matrix V is considered. Hence, the vectors f and v are

$$f^T = \begin{pmatrix} \lambda s_y + e_y\left(\alpha_y d_{2y} + \alpha_o d_{2o}\right) \\ p_y\sigma_y e_y + a_y\left(\alpha_y d_{2y} + \alpha_o d_{2o}\right) \\ \left(1-p_y\right)\sigma_y e_y + d_{1y}\left(\alpha_y d_{2y} + \alpha_o d_{2o}\right) \\ \left(1-\chi_y\right)\gamma_y a_y + m_y\gamma_{1y}d_{1y} + \xi_y q_{3y} + q_{2y}\left(\alpha_y d_{2y} + \alpha_o d_{2o}\right) \\ \lambda\psi s_o + e_o\left(\alpha_y d_{2y} + \alpha_o d_{2o}\right) \\ p_o\sigma_o e_o + a_o\left(\alpha_y d_{2y} + \alpha_o d_{2o}\right) \\ \left(1-p_o\right)\sigma_o e_o + d_{1o}\left(\alpha_y d_{2y} + \alpha_o d_{2o}\right) \\ \left(1-\chi_o\right)\gamma_o a_o + m_o\gamma_{1o}d_{1o} + \xi_o q_{3o} + q_{2o}\left(\alpha_y d_{2y} + \alpha_o d_{2o}\right) \end{pmatrix} \tag{A.10}$$

and

$$v^T = \begin{pmatrix} \left(\sigma_y + \phi\right)e_y \\ \left(\gamma_y + \eta_y + \chi_y + \phi\right)a_y \\ \left(\gamma_{1y} + \eta_{1y} + \phi\right)d_{1y} \\ \left(\gamma_{3y} + \eta_{2y} + \varpi_y + \phi\right)q_{2y} \\ \left(\sigma_o + \phi\right)e_o \\ \left(\gamma_o + \eta_o + \chi_o + \phi\right)a_o \\ \left(\gamma_{1o} + \eta_{1o} + \phi\right)d_{1o} \\ \left(\gamma_{3o} + \eta_{2o} + \varpi_o + \phi\right)q_{2o} \end{pmatrix}, \tag{A.11}$$

where the superscript T stands for the transposition of a matrix, from which we obtain the matrices F and V (see [3]) evaluated at the trivial equilibrium P^0, which were

omitted. The next generation matrix FV^{-1} is

$$FV^{-1} =$$

$$\begin{bmatrix}
0 & \frac{\frac{\varepsilon}{\omega}\beta_{1y}s_y^0}{\gamma_y+\eta_y+\chi_y+\phi} & \frac{\frac{\varepsilon}{\omega}\beta_{2y}s_y^0}{\gamma_{1y}+\eta_{1y}+\phi} & \frac{\frac{\varepsilon}{\omega}\beta_{3y}z_y s_y^0}{\gamma_{3y}+\eta_{2y}+\varpi_y+\phi} & 0 & \frac{\frac{\varepsilon}{\omega}\beta_{1o}s_y^0}{\gamma_o+\eta_o+\chi_o+\phi} & \frac{\frac{\varepsilon}{\omega}\beta_{2o}s_y^0}{\gamma_{1o}+\eta_{1o}+\phi} & \frac{\frac{\varepsilon}{\omega}\beta_{3o}z_o s_y^0}{\gamma_{3o}+\eta_{2o}+\varpi_o+\phi} \\
\frac{p_y\sigma_y}{\sigma_y+\phi} & 0 & 0 & 0 & 0 & 0 & 0 & 0 \\
\frac{(1-p_y)\sigma_y}{\sigma_y+\phi} & 0 & 0 & 0 & 0 & 0 & 0 & 0 \\
0 & \frac{(1-\chi_y)\gamma_y}{\gamma_y+\eta_y+\phi} & \frac{m_y\gamma_{1y}}{\gamma_{1y}+\eta_{1y}+\phi} & 0 & 0 & 0 & 0 & 0 \\
0 & \frac{\frac{\varepsilon}{\omega}\beta_{1y}\psi s_o^0}{\gamma_y+\eta_y+\chi_y+\phi} & \frac{\frac{\varepsilon}{\omega}\beta_{2y}\psi s_o^0}{\gamma_{1y}+\eta_{1y}+\phi} & \frac{\frac{\varepsilon}{\omega}\beta_{3y}z_y\psi s_o^0}{\gamma_{3y}+\eta_{2y}+\varpi_y+\phi} & 0 & \frac{\frac{\varepsilon}{\omega}\beta_{1o}\psi s_o^0}{\gamma_o+\eta_o+\chi_o+\phi} & \frac{\frac{\varepsilon}{\omega}\beta_{2o}\psi s_o^0}{\gamma_{1o}+\eta_{1o}+\phi} & \frac{\frac{\varepsilon}{\omega}\beta_{3o}z_o\psi s_o^0}{\gamma_{3o}+\eta_{2o}+\varpi_o+\phi} \\
0 & 0 & 0 & 0 & \frac{p_o\sigma_o}{\sigma_o+\phi} & 0 & 0 & 0 \\
0 & 0 & 0 & 0 & \frac{(1-p_o)\sigma_o}{\sigma_o+\phi} & 0 & 0 & 0 \\
0 & 0 & 0 & 0 & 0 & \frac{(1-\chi_o)\gamma_o}{\gamma_o+\eta_o+\phi} & \frac{m_o\gamma_{1o}}{\gamma_{1o}+\eta_{1o}+\phi} & 0
\end{bmatrix}$$

and the corresponding characteristic equation FV^{-1} is

$$\varkappa^3\left[\varkappa^3 - \frac{\varepsilon}{\omega}\left(R_{1y}s_y^0 + R_{1o}s_o^0\right)\varkappa - \frac{\varepsilon}{\omega}\left(R_{2y}s_y^0 + R_{2o}s_o^0\right)\right] = 0, \tag{A.12}$$

where the partially reduced reproduction numbers R_{1y}, R_{2y}, R_{1o}, and R_{2o} are

$$\left\{\begin{array}{lcl}
R_{1y} & = & p_y\frac{\sigma_y}{\sigma_y+\phi}\frac{\beta_{1y}}{\gamma_y+\eta_y+\phi} + (1-p_y)\frac{\sigma_y}{\sigma_y+\phi}\frac{\beta_{2y}}{\gamma_{1y}+\eta_{1y}+\phi} \\
R_{1o} & = & p_o\frac{\sigma_o}{\sigma_o+\phi}\frac{\beta_{1o}\psi}{\gamma_o+\eta_o+\phi} + (1-p_o)\frac{\sigma_o}{\sigma_o+\phi}\frac{\beta_{2o}\psi}{\gamma_{1o}+\eta_{1o}+\phi} \\
R_{2y} & = & \left[p_y\frac{\sigma_y}{\sigma_y+\phi}(1-\chi_y)\frac{\gamma_y}{\gamma_y+\eta_y+\phi} + (1-p_y)\frac{\sigma_y}{\sigma_y+\phi}m_y\frac{\gamma_{1y}}{\gamma_{1y}+\eta_{1y}+\phi}\right]\times \\
 & & \frac{z_y\beta_{3y}}{\gamma_{3y}+\eta_{2y}+\varpi_y+\phi} \\
R_{2o} & = & \left[p_o\frac{\sigma_o}{\sigma_o+\phi}(1-\chi_o)\frac{\gamma_o}{\gamma_o+\eta_o+\phi} + (1-p_o)\frac{\sigma_o}{\sigma_o+\phi}m_o\frac{\gamma_{1o}}{\gamma_{1o}+\eta_{1o}+\phi}\right]\times \\
 & & \frac{z_o\beta_{3o}\psi}{\gamma_{3o}+\eta_{2o}+\varpi_o+\phi}.
\end{array}\right. \tag{A.13}$$

The spectral radius $\rho\left(FV^{-1}\right)$is the biggest solution of a third degree polynomial, which is not easy to evaluate. The procedure proposed in [4] allows us to obtain the threshold R_c as the sum of coefficients of the characteristic equation, where R_c is the reduced reproduction number given by

$$R_c = R_{cy} + R_{co}, \quad \text{where} \quad \left\{\begin{array}{l} R_{cy} = R_y s_y^0, \\ R_{co} = R_o s_o^0, \end{array}\right. \quad \text{with} \quad \left\{\begin{array}{l} R_y = \frac{\varepsilon}{\omega}\left(R_{1y} + R_{2y}\right), \\ R_o = \frac{\varepsilon}{\omega}\left(R_{1o} + R_{2o}\right). \end{array}\right. \tag{A.14}$$

Hence, the trivial equilibrium point P^0 is locally asymptotically stable if $R_c < 1$.

The basic reproduction number R_0 is given by

$$R_0 = R_{0y} + R_{0o}, \quad \text{where} \quad \left\{\begin{array}{l} R_{0y} = R_y s_y^0, \\ R_{0o} = R_o s_o^0, \end{array}\right. \quad \text{with} \quad \left\{\begin{array}{l} R_y = R_{1y} + R_{2y}, \\ R_o = R_{1o} + R_{2o}, \end{array}\right. \tag{A.15}$$

where R_{1y}, R_{2y}, R_{1o}, and R_{2o} are obtained from equation (A.13) letting $\varepsilon = \omega = 1$ (absence of protective measures), $\eta_j = \eta_{1j} = \eta_{2j} = 0$ (absence of tests), and $\varpi_j = 0$ (absence of educational campaign), for $j = y, o$. All these control actions to decrease the transmission are absent at the beginning of the epidemic, and the definition of the basic reproduction number is fulfilled.

The basic reproduction number R_0 is the secondary cases produced by one infectious person (could be anyone in one of the classes harboring virus) in a completely

susceptible young and elder populations without constraints [1]. Let us understand R_{1j} and R_{2j}, $j = y, o$, stressing that the interpretation is the same for both subpopulations; hence we drop out subscript j. To facilitate the understanding, we consider this infectious person in exposed class E. This person enters into one of the contagious classes composed of asymptomatic (A), pre-diseased (D_1), and a fraction of mild CoViD-19 (Q_2).

1. R_1 takes into account the transmission by one person in the asymptomatic A or pre-diseased D_1 class. We interpret for asymptomatic person transmitting (between parentheses, for pre-diseased person) infection. One infectious person survives during the incubation period with probability $\sigma/\left(\sigma+\phi\right)$ and enters into asymptomatic class with probability p (pre-diseased, with $1-p$) and generates, during the time $1/\left(\gamma+\phi\right)$ (pre-diseased, $1/\left(\gamma_1+\phi\right)$) staying in this class, on average $\beta_1/\left(\gamma+\phi\right)$ (pre-diseased, $\beta_2/\left(\gamma_1+\phi\right)$) secondary cases.

2. R_2 takes into account the transmission by a mild CoViD-19 person. An infectious person has two routes to reach Q_2: passing through A or D_1 (this case is given between parentheses). One infectious person survives during the incubation period with probability $\sigma/\left(\sigma+\phi\right)$ and enters into asymptomatic (pre-diseased) class with probability p (pre-diseased, with $1-p$); survives in this class and also is not caught by a test with probability $\gamma/\left(\gamma+\phi\right)$ (pre-diseased, $\gamma_1/\left(\gamma_1+\phi\right)$) and enters into mild CoViD-19 class Q_2 with probability $1-\chi$ (pre-diseased, m); and generates, during the time $1/\left(\gamma_3+\phi\right)$ staying in this class, on average $z\beta_3/\left(\gamma_3+\phi\right)$ secondary cases.

Hence, $R_0 = R_{0y}s_y^0 + R_{0o}s_o^0$ is the overall number of secondary cases generated by one primary case introduced into a completely susceptible young and elder subpopulations. The model parameters are not accurate, and it is expected that the inaccuracy of those values influence R_0. The sensitivity analysis can assess the variation of R_0 with uncertainties in the parameters (see below).

A.2.2 The effective reproduction number R_{ef} – The non-trivial equilibrium point

The effective reproduction number R_{ef} is defined by one of the dynamic system equations at the non-trivial equilibrium point P^*. For instance, for the SEIR model, the equation relating susceptible persons must obey at the equilibrium, $R_0 s = 1$, from which R_{ef} is defined by $R_{ef} = R_0 s$, where s varies with time. Notice that at the beginning of the epidemic ($t = 0$) we have $s = 1$, and $R_{ef} = R_0$, and at the steady-state ($t \to \infty$), $s = s^*$ and obeys $R_{ef} = 1$, resulting in $s^* = 1/R_0$. In the preceding section, we showed that R_0 is given by Eq (A.15). Still, we did not prove that $s^* = 1/R_0$ due to the complexity of the new system of Eqs (A.6), (A.7) and (A.8), and it is not an easy task to determine the non-trivial (endemic) equilibrium point P^*.

However, when $z_y = z_o = 0$, we can show that the inverse of the basic reproduction number R_0 is the fraction of susceptible persons in the steady-state [6]. However, we have young and elder subpopulations; hence the fraction of susceptible individuals at endemic equilibrium $s^* = s_y^* + s_o^*$ is related generically as

$$f\left(s_y^*, s_o^*\right) = \frac{1}{R_0} = \frac{1}{R_{0y}s_y^0 + R_{0o}s_o^0}, \tag{A.16}$$

and the effective reproduction number R_{ef} [7], which varies with time, can not be defined neither by $R_{ef} = R_0\left(s_y + s_o\right)$ nor $R_{ef} = R_{0y}s_y + R_{0o}s_o$. For instance, for dengue transmission model, $f\left(s_1^*, s_2^*\right) = s_1^* \times s_2^*$, where s_1^* and s_2^* are the fractions at

equilibrium of, respectively, humans and mosquitoes [8]. For tuberculosis model considering drug-sensitive and resistant strains, s^* is solution of a second degree polynomial [9]. For this reason, we define the approximated effective reproduction number R_{ef} as

$$R_{ef} \approx R_y s_y + R_o s_o, \tag{A.17}$$

which depends on time, and when it attains steady-state ($R_{ef} = 1$), we have $s^* = 1/R_0$.

The basic reproduction number R_0 obtained from mathematical modelings provides two useful information: At the beginning of the epidemic ($t = 0$), R_0 gives the magnitude of control efforts, and when the epidemic reaches the steady-state (after many waves of the epidemic, that is, $t \to \infty$), R_0 measures its severity providing the fraction of susceptible individuals, in general, $s^* = 1/R_0$ [9]. Between these two extremes, the effective reproduction number R_{ef} dictates the course of an epidemic, which follows decaying oscillations around $R_{ef} = 1$ [10].

The approximate effective reproduction number R_{ef}, given by Eq (A.17), can be applied directly to the system of Eqs (A.2), (A.3), and (A.4) by substituting s_y and s_o by S_y/N and S_o/N, that is,

$$R_{ef} = \frac{\varepsilon}{\omega}\left[(R_{1y} + R_{2y})\frac{S_y}{N} + (R_{1o} + R_{2o})\frac{S_o}{N}\right], \tag{A.18}$$

where S_y, S_o, and N vary with time (remember that the fractions S_y/N and S_o/N approach steady-state). We describe the variation of R_{ef} during the epidemic.

1. Natural epidemic. The population, initially, did not adopt any protection measures against the CoViD-19 outbreak, and at $t = 0$ we have $R_{ef}(0) = R_0$, where the basic reproduction number is obtained by substituting s_y^0 and s_o^0 by N_{0y}/N_0 and N_{0o}/N_0 in Eq (A.9), resulting in

$$R_0 = R_y\frac{N_{0y}}{N_0} + R_o\frac{N_{0o}}{N_0} = (R_{1y} + R_{2y})\frac{N_{0y}}{N_0} + (R_{1o} + R_{2o})\frac{N_{0o}}{N_0}, \tag{A.19}$$

   where R_y and R_o are given by Eq (A.15). For $t > 0$, R_{ef} decreases as susceptible populations decrease.

2. Isolation. At $t = \tau^{is}$ a pulse in isolation is introduced, decreasing the number of susceptible persons, from $S_y\left(\tau^{is-}\right)$ and $S_o\left(\tau^{is-}\right)$ to $S_y\left(\tau^{is+}\right)$ and $S_o\left(\tau^{is+}\right)$, see Eqs (6) and (7) in the main text. The decrease in the susceptible populations at τ^{is} results in $R_{ef}(\tau^{is-})$ jumping down to $R_{ef}(\tau^{is+}) = R_r$, where the reduced reproduction number R_r is given by

$$R_r = (R_{1y} + R_{2y})\frac{S_y\left(\tau^{is-}\right)(1 - k_y)}{N\left(\tau^{is}\right)} + (R_{1o} + R_{2o})\frac{S_o\left(\tau^{is-}\right)(1 - k_o)}{N\left(\tau^{is}\right)}, \tag{A.20}$$

   where R_{1y}, R_{2y}, R_{1o}, and R_{2o} are given by Eq (A.15). For $t > \tau^{is}$, R_{ef} decreases as susceptible populations decrease.

3. Adopting protective measures. The protective measures are incorporated in the modeling by the factor ε in the circulating population and the restricted contact by ω in the isolated population. At the time of introducing these measures T, the effective reproduction number just before the time of the adoption of protective measures $R_{ef}(T^-)$ jumps down to $R_{ef}(T^+) = R_p$ by factor ε/ω, that is,

$$R_p = \frac{\varepsilon}{\omega}\left[(R_{1y} + R_{2y})\frac{S_y\left(T^-\right)}{N\left(T^-\right)} + (R_{1o} + R_{2o})\frac{S_o\left(T^-\right)}{N\left(T^-\right)}\right], \tag{A.21}$$

   where R_{1y}, R_{2y}, R_{1o}, and R_{2o} are given by Eq (A.15). For $t > T$, R_{ef} decreases as susceptible populations decrease.

Therefore, the importance of a mathematical model is the capability of providing the basic and effective reproduction numbers, which give information about the risk of infection during the epidemic. Our model is complex, but we could obtain R_0, which was not an easy task. However, a more complex model can be formulated, incorporating novel findings of the CoViD-19 epidemic, but we must be aware that the basic reproduction number must be obtained from the model.

A.3 The sensitivity analysis of R_0

We perform the sensitivity analysis of R_0 following Yang [11]. The basic reproduction number $R_0 = R_{0y}(\Theta_y) + R_{0o}(\Theta_o)$ depends on the parameters set $\Theta = \Theta_y + \Theta_o$, with Θ_j, $j = y, o$, given by

$$\Theta_j = \{\sigma_y, \gamma_j, \gamma_{1j}, \gamma_{3j}, \beta_{1j}, \beta_{2j}, \beta_{3j}, \chi_j, p_j, m_j, z_j\},$$

where $R_{0y} = (R_{1y} + R_{2y}) s_y^0$ and $R_{0o} = (R_{1o} + R_{2o}) s_o^0$, with R_{1y}, R_{2y}, R_{1o}, and R_{2o} given by Eq (A.13) letting $\eta_j = \eta_{1j} = \eta_{2j} = 0$ (absence of tests) and $\varpi_o = 0$ (absence of educational campaign), for $j = y, o$. We have 11 parameters, each one represented by u_i, $i = 1, \cdots, 11$. The quite invariant birth rate ϕ and the aging rate (φ) in the fraction of susceptible persons are not considered in the sensitivity analysis. Hence, s_y^0 and s_o^0 are invariant.

Let us assume that each random parameter $u_{ji} \in \Theta_j$, with $j = y, o$ and $i = 1, \cdots, 11$, is estimated with error Δu_{ji}. The quadratic error $\varrho^2_{R_{0j}}$ of R_{0j} is the sum of each parameters' error defined by

$$\varrho^2_{R_{0j}} \approx H_j^T V_{\Theta_j} H_j,$$

where H_j is the sensitivity matrix 11×1, with each element H_{ji} given by

$$H_{ji} = \frac{\partial R_{0j}}{\partial u_{ji}}, \tag{A.22}$$

and V_{Θ_j} is the covariance matrix of parameters [12]. Notice that the sensitivity of each parameter $H_{ji} = \partial R_{0j}/\partial u_{ji}$ of R_{0j} assumes real values. If $H_{ji} < 0$, the parameter u_{ji} contributes to decrease R_{0j}. We evaluate the partial derivative $H_{ji} = \partial R_{0j}/\partial u_{ji}$ at the mean values of u_j given in Table 2 in the main text. If we assume that the parameters have independent distributions, the covariances are zero (V_{Θ_j} is diagonally composed by Δu_{ji}), then the error $\varrho_{R_{0j}}$ is approximated by

$$\varrho_{R_{0j}} \approx \sqrt{\sum_{i=1}^{11} \varrho^2_{u_{ji}}} = \sqrt{\sum_{i=1}^{11} \left(\frac{\partial R_{0j}}{\partial u_{ji}} \Delta u_{ji}\right)^2} = \sqrt{\sum_{i=1}^{11} (H_{ji} \Delta u_{ji})^2}. \tag{A.23}$$

An alternative approach to perform the sensitivity analysis is considering the basic reproduction number R_0 as a function of the variables $u_{ji} \in \Theta_j$, with $j = y, o$ and $i = 1, \cdots, 11$. The first-order approximation of the function R_{0j} with respect to the variable u_{ji} varying in $\Delta u_{ji} > 0$ is given by

$$\Delta R^i_{0j} = R_{0j}(u_{ji} \pm \Delta u_{ji}) - R_{0j}(u_{ji}) \approx \pm \frac{\partial R_{0j}}{\partial u_{ji}} \Delta u_{ji}.$$

Instead of ΔR^i_{0j}, we evaluate the quadratic variation $\left(\Delta R^i_{0j}\right)^2$ for each variable u_{ji}. The sum of the quadratic variations is given by

$$\Delta R^2_{0j} \approx \sum_{i=1}^{11} \left(\frac{\partial R_{0j}}{\partial u_{ji}} \Delta u_{ji}\right)^2, \tag{A.24}$$

which is equal to $\varrho^2_{R_{0j}}$ obtained by the previous approach.

A.4 Remarks about the general model

The model given by the system of Eqs (A.2), (A.3), and (A.4) is non-autonomous and non-constant deterministic model. This deterministic model provides an overall view of the CoViD-19 epidemic. However, some particular/specific features of the epidemic can be dealt with.

In our model, we can incorporate the mobility of infectious individuals and apply the technique of finding traveling waves. We can achieve the geographic expansion of SARS-CoV-2 by calculating the velocity of the front of the wave. For instance, the dengue expansion in São Paulo State [13] and West Nile Virus in American Continent [14] were evaluated by the method of finding traveling waves.

We did not consider uncertainties in our model parameters, which can be introduced considering the stochastic version. For instance, the transmission rate βdt can be written as $\bar{\beta} dt \pm \sigma_{\bar{\beta}} dB(t)$, where $\sigma_{\bar{\beta}}$ is the standard deviation of average $\bar{\beta}$ and B is the Brownian motion. Gray *et al.* [15] analyzed the SIS model to determine the standard deviation $\sigma_{\bar{\beta}}$ and performed computational simulations.

Instead of continuously varying the number of individuals in each class, our model can be simulated, allowing integer variation in the population. For instance, Freitas [16] used a continuous-time stochastic version of the SEIRV (V stands for vaccinated subpopulation) model with integer-valued states. The simulations were done applying the Gillespie algorithm, considering that the transitions between compartments occur randomly according to a Markov chain with transition rates equal to the deterministic rates. A more complex stochastic process can also be applied considering all classes in our model as stochastic variables. For instance, the stochastic process was applied to schistosomiasis transmission [17]. However, in the stochastic process, the average values can be calculated for the stochastic variables, and, in some cases, these averaged variables follow the corresponding deterministic model.

Computational modelings can be formulated. There are innumerous methods, being the agent-based model (ABM) the most widespread. ABM is derived from cellular automata letting each cell carrying on much information. For instance, Ferreira *et al.* [18] applied cellular automata approach to evaluate the sterile insect technique's suitability to control *Aedes aegipty* when breeding sites are homogeneously and heterogeneously distributed. However, in computational models, after obtaining thousands of trajectories, the mean-field is calculated.

B Description of data collection from São Paulo State and Spain

We first present a detailed description of the data collected from São Paulo State, and briefly the data collected from Spain.

B.1 São Paulo State

Fig B.1 shows the daily (a) and accumulated (b) severe CoViD-19 cases, and daily (c) and accumulated (d) deaths due to CoViD-19 collected from São Paulo State [19]. In Fig B.1(b), A indicates the time at which isolation was introduced, and B represents the time at which the upward concavity changed to downward concavity (inflection point, which occurs when the daily cases reach the maximum value). In São Paulo State, the isolation was introduced on March 24 (A), and the inflection point occurred after 80 *days* on June 12 (B).

Fig B.2 shows the population's proportion in isolation (a), and daily cases plus the proportion in isolation moved 9 days to the right (b). The horizontal line in Fig B.2(a)

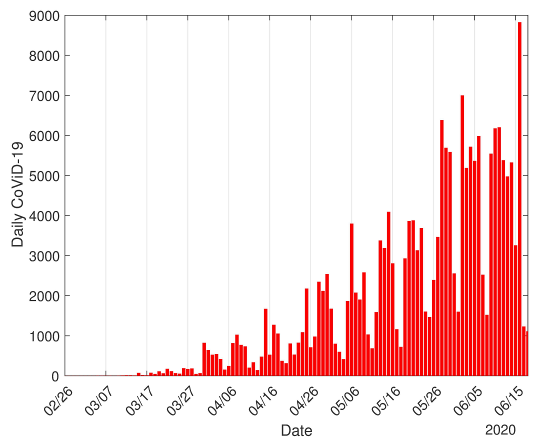

9000
8000
7000
6000
5000
4000
3000
2000
1000
0
Daily CoViD-19
02/26
03/07
03/17
03/27
04/06
04/16
04/26
05/06
05/16
05/26
06/05
06/15
Date
2020

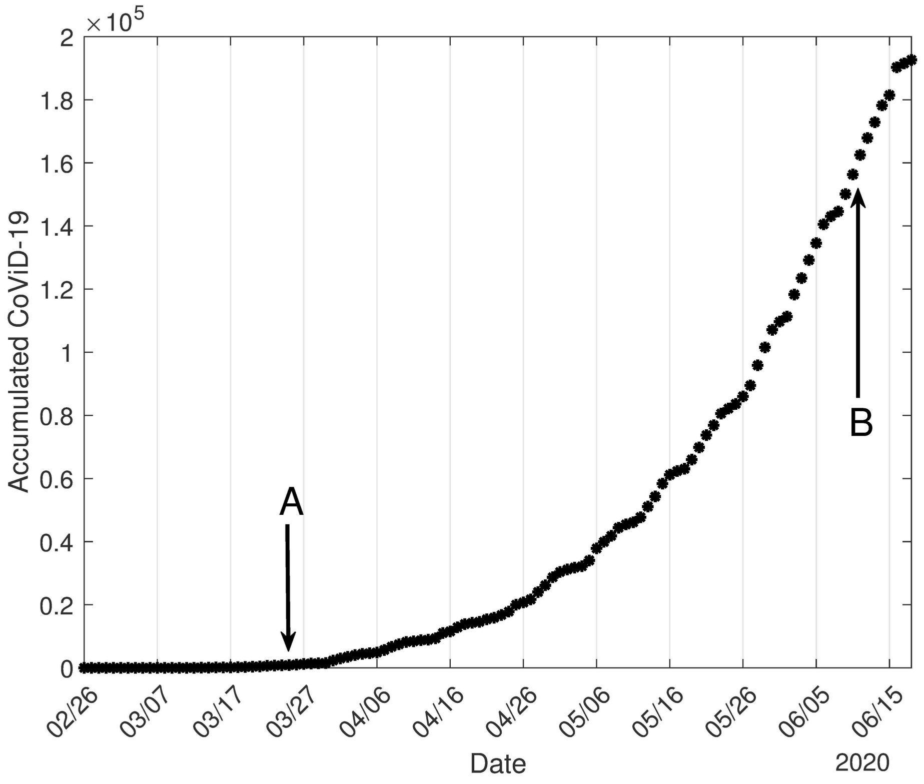

× 10^5
2
1.8
1.6
1.4
1.2
1
0.8
0.6
0.4
0.2
0
Accumulated CoViD-19
A
B
02/26
03/07
03/17
03/27
04/06
04/16
04/26
05/06
05/16
05/26
06/05
06/15
Date
2020

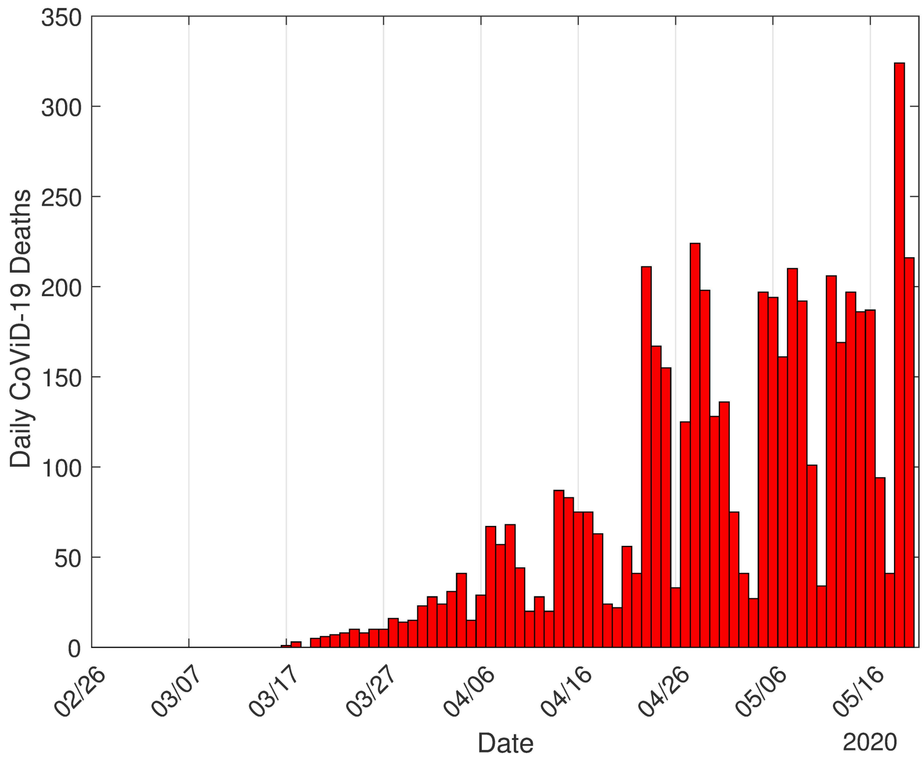

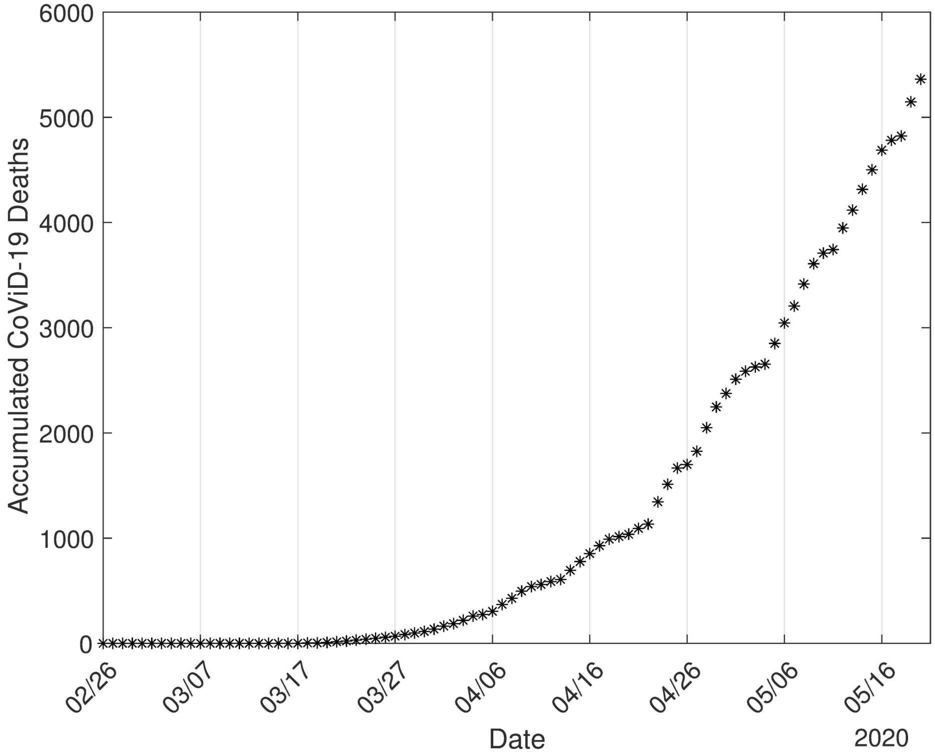

Fig B.1. The daily (a) and accumulated (b) covid-19 cases, and daily (c) and accumulated (d) deaths, using covid-19 cases collected in São Paulo State. In (b), A indicates the time at which isolation was introduced, and B represents the time at which inflection point occurred.

corresponds to the mean value $k_{mean} = 0.53$, around which daily proportions vary impacting the transmission [20].

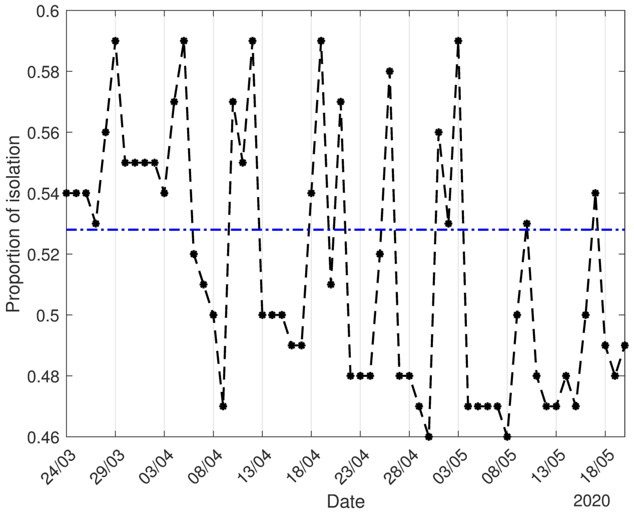

Proportion of isolation
Date
2020

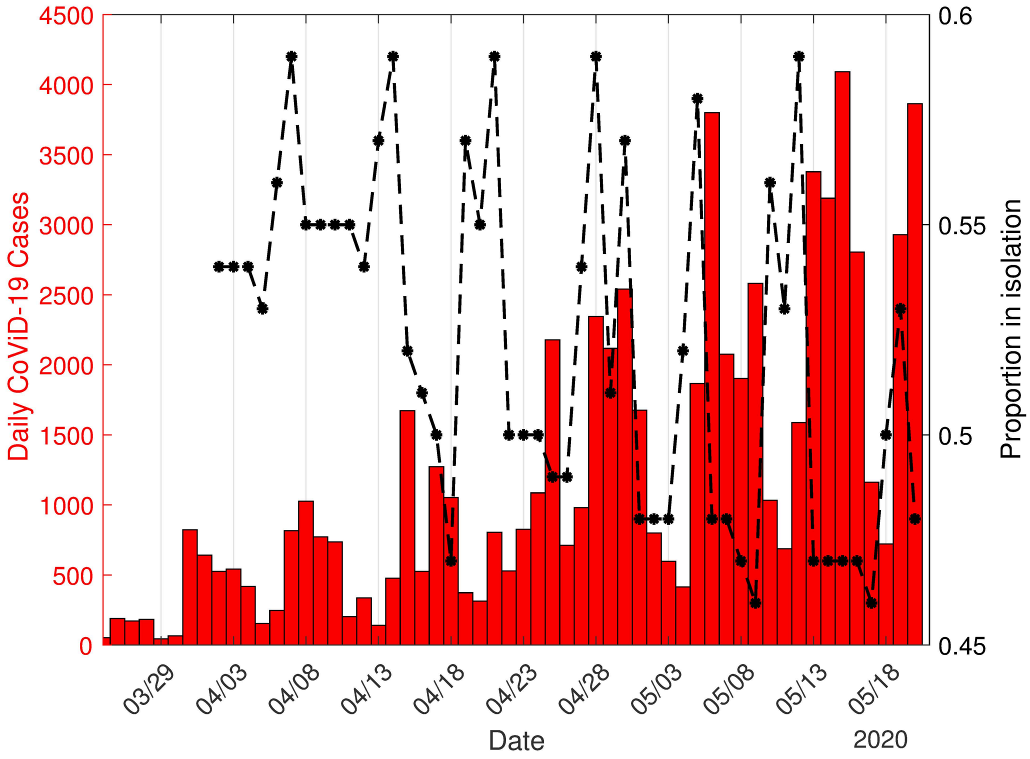

Daily CoViD-19 Cases
Proportion in isolation
Date
2020

Fig B.2. The proportion in isolation in São Paulo State (a), and daily cases plus the proportions in isolation moved 9 days to the right (b).

We discuss roughly the collected CoViD-19 cases shown in Fig B.1. Interestingly, Fig B.1(a) shows that the daily data present weekly seasonality, with lower cases at the weekend [19], due to the procedure of registering the day at which occurred the confirmation by laboratory testing, not the beginning of the symptoms.

(A) The number of SARS in São Paulo State registered in the site of Ministry of Health (Brazil) [21] shows increasing beyond the average cases occurred in past years since March 8, 2020 (around $1,000$ cases in the 11th epidemiological week (hereafter, week), March 8-14), and reach peak 2 weeks later (around $4,000$ cases in the 13th week, March 22-28). After this epidemiological week, the notification as SARS initiates a decreasing trend, maybe due to increased testing of severe CoViD-19 cases (on March 31, there were 822 cases, but one day earlier, only 66 cases, and around 180 cases per day in the 13th week). Fig B.1(b) shows this jump up on March 31. This increased number of cases should be explained by more testing among SARS to identify CoViD-19, or by the exponential-like increase of the epidemic in the beginning, or probably by both. Fig B.1(a) shows an unusual jump up when comparing 13th week and 14th week (March 29 - April 4), which is not observed in the next weeks, suggesting that the isolation decreased the force of infection. Indeed, the isolation was introduced on March 24, but after 10 days, on April 3, there is a change in the exponential-like trend, becoming less abrupt. Fig B.1(b) shows an increasing trend in blocks of week affected by weekly seasonality shown in Fig B.1(a) depending on the proportion in isolation that occurred 9 or 10 days earlier.

(B) Let us compare severe CoViD-19 cases and proportion in isolation week by week, Fig B.1(b). Notice that there is a jump up from the 13th to 14th week, showing an exponential-like increase. However, there is not jump in the 15th week (April 5-11), possibly indicating the effects of isolation. In the 16th week (April 12-18), there were substantial variations in the CoViD-19 cases, maybe due to huge variation in the proportions in isolation 9 days earlier. In the 17th week (April 19-25), the increased number of cases corresponds to decreased proportions in isolation, including weekend (on April 25, Sunday, there was the highest number of cases). This increasing trend continued in the next 18th week (April 26 - May 2), when the proportions in isolation fluctuated, but a relatively small number of cases were registered during the extended holiday (May 1-3). The behavior observed in the 17th and 18th weeks may be the effects of manifestation against isolation occurred on April 18: the peak on April 25 and the high number of cases lasting until April 30, that is, 7 to 12 days after the manifestation is the interval with median 9.5 and variation 2.5 (sum of incubation and pre-diseased periods is 9.8).

(C) From Fig B.1(b), the accumulated CoViD-19 cases show three periods with different trends. The first period from February 26 to April 3 corresponds to the natural epidemic. The second period, from April 4 to April 12, corresponds to the isolation effectively decreasing the epidemic. In the last period, since April 13, the additional reduction in the transmission of CoViD-19 occurs due to the protective measures.

From the daily registered deaths due to CoViD-19 shown in Fig B.1, we observe a correspondence between the number of fatalities, Fig B.1(c), and the number of CoViD-19 cases, Fig B.1(a), occurred around 15 days ago. For instance, on May 18, São Paulo State registered 41 deaths, and on May 19, 324. However, on May 3 and 4, 15 and 14 days before May 18, there were recorded, respectively, 598 and 415 CoViD-19 cases, while on May 5 and 6, 14 and 13 days before May 19, $1,866$ and $3,800$ CoViD-19 cases were registered. Hence, we can infer that the number of deaths corresponds to cases of CoViD-19 that occurred around 14 to 15 days earlier. For instance, on May 13, 14, 15, and 16, the daily registered CoViD-19 cases were, respectively, $3,378$, $3,189$, $4,092$, and $2,805$, and it is expected a high number of deaths after 15 days.

B.2 Spain

Fig B.3 shows the daily (a) and accumulated (b) severe CoViD-19 cases, and daily (c) and accumulated (d) deaths due to CoViD-19 collected from Spain, from January 31 to May 20 [22]. In Fig B.3(b), A indicates the time at which lockdown was introduced, and B represents the time at which the inflection point occurred. In Spain, the lockdown was introduced on March 16 (A), and the inflection point occurred after 11 *days* on March 27 (B).

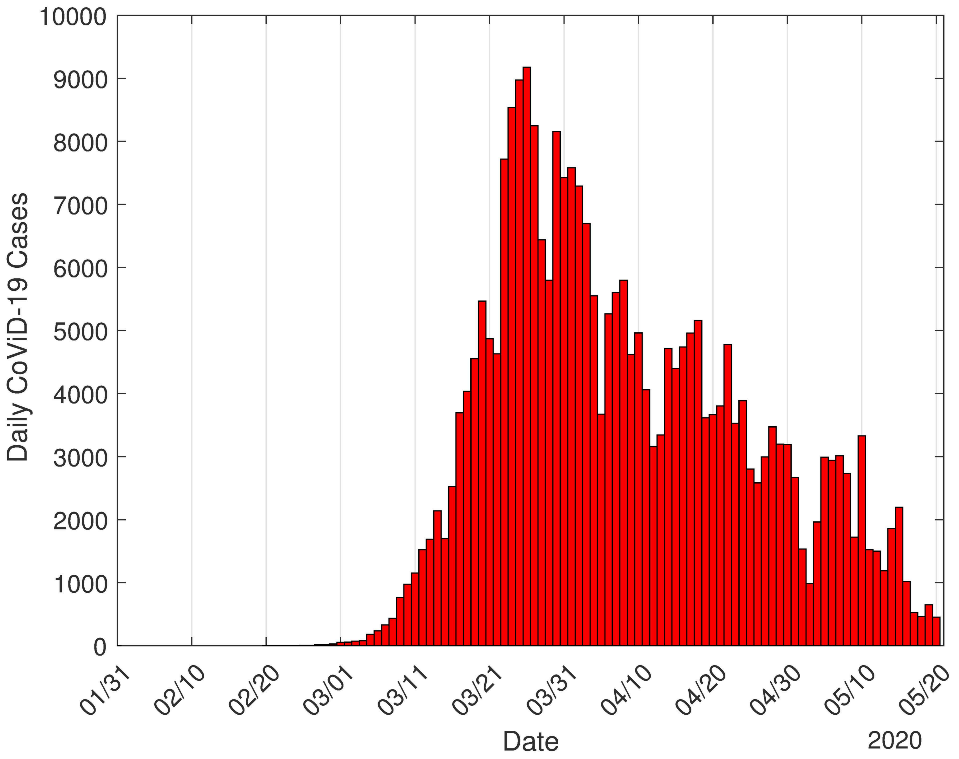

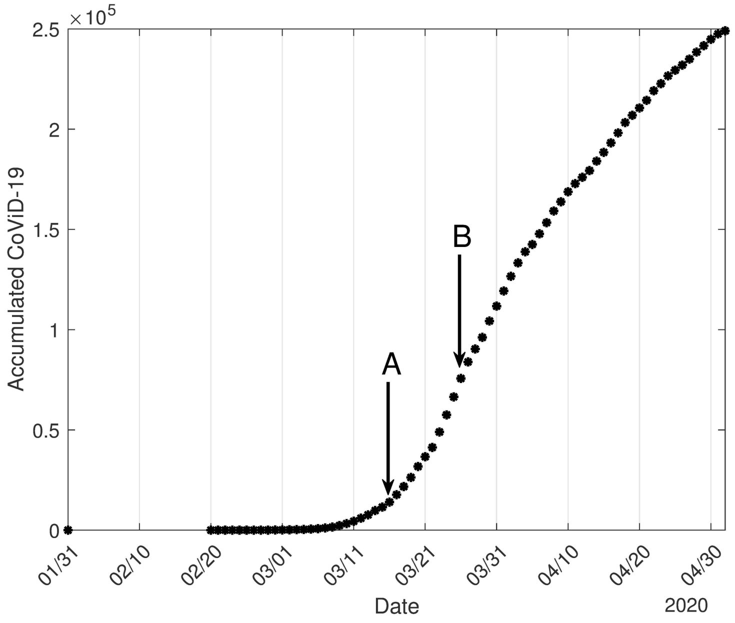

×10^5
2.5
2
1.5
1
0.5
0
Accumulated CoViD-19
A
B
01/31
02/10
02/20
03/01
03/11
03/21
03/31
04/10
04/20
04/30
Date
2020

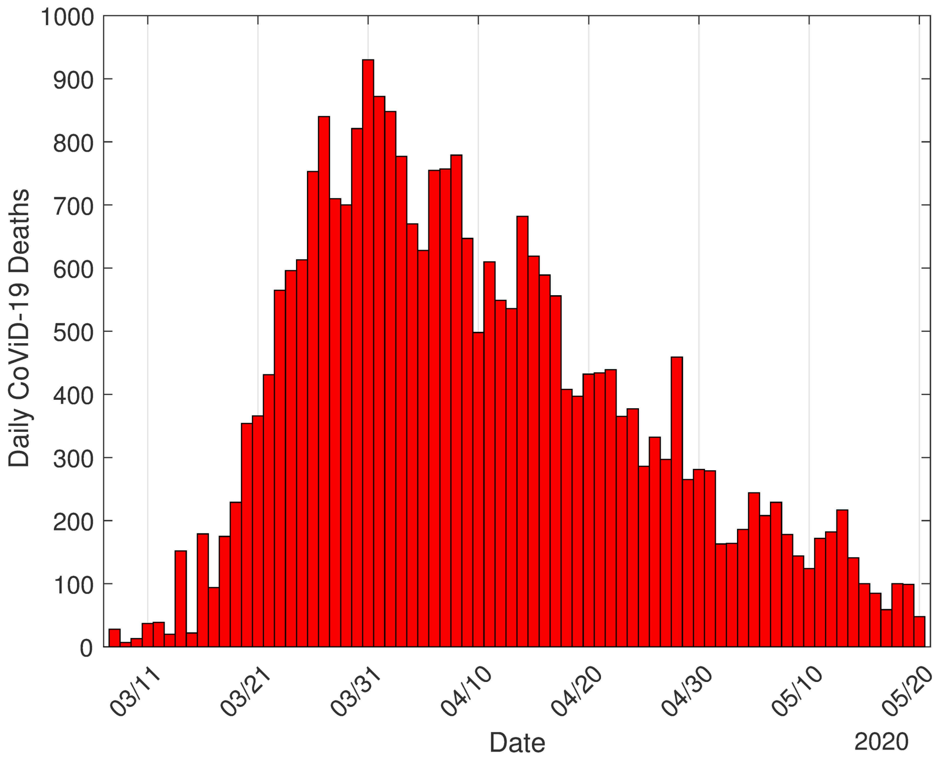

1000
900
800
700
600
500
400
300
200
100
0
Daily CoViD-19 Deaths
03/11
03/21
03/31
04/10
04/20
04/30
05/10
05/20
Date
2020

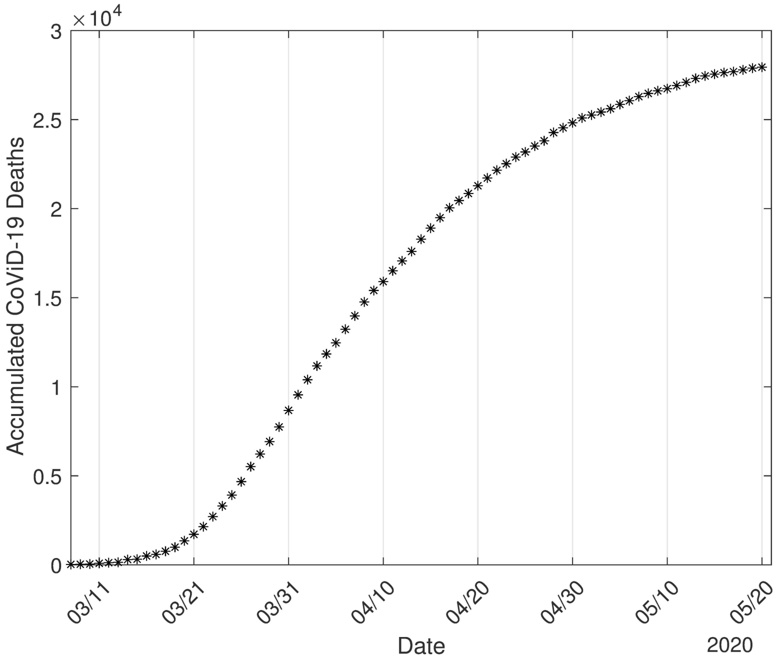

Fig B.3. The daily (a) and accumulated (b) covid-19 cases, and daily (c) and accumulated (d) deaths, using covid-19 cases collected in Spain. In (b), A indicates the time at which isolation was introduced, and B represents the time at which inflection point occurred.

Analyzing the data collected from Spain, we observe three trends. From January 31 to March 21, the first period had an exponential-like increase, which is followed by the second period with less increase from March 22 to 28, and the last period with a slow rise since March 29. The effects of lockdown implemented on March 16 are expected to appear 9 days later [23], on March 25. The intermediate period from March 22 to 28 is centered on March 25 with 3 days of variation, called the transition period from natural to lockdown epidemic. Hence, we divide the epidemiological scenarios of Spain into three stages, and we estimate the transmission rates β_y and β_o, the protection factor ε, and the decreasing factor ω. The additional mortality rates α_y and α_o are also estimated.

C Model parameters evaluation

We present the model parameters' estimation based on the collections of data from São Paulo State and Spain. In both regions, we consider the three periods presenting different trends in the number of accumulated CoViD-19 cases. The solutions of the system of Eqs (1), (2) and (3) are obtained numerically using the initial conditions given by Eq (D.3).

C.1 São Paulo State

Using data collected in São Paulo State from February 26 to May 7, we evaluate the transmission rates (β_y and β_o), the proportion in the isolated population (k), reduction in the transmission rates due to the protective measures adopted by the circulating population (ε), and the additional mortality rates (α_y and α_o).

C.1.1 Natural epidemic – Evaluating the transmission rates

The effects of isolation implemented on March 24 are expected to appear later on the daily registered cases of severe CoViD-19 (the sum of incubation and pre-diseased infection periods (see Table 2) is 9.8 days). Hence, the data from February 26 to April 3 of severe CoViD-19 cases portray the natural epidemic; that is, the transmission of infection is occurring without any kind of intervention. This epidemiological scenario fulfills the definition of the basic reproduction number R_0 (entire population is susceptible in the absence of constraints), allowing its estimation.

We evaluate the transmission rates taking into account the confirmed cases from February 26 (t_1) to April 3 (t_{38}), and using Eq (D.1). The evaluated values are $\beta_y = 0.78$ and $\beta_o = 0.90$ (both in $days^{-1}$), where $\psi = 1.15$, resulting in the basic reproduction number $R_0 = 9.24$ (partials $R_{0y} = 7.73$ and $R_{0o} = 1.51$), according to Eq (A.19).

Fig C.1(a) shows the estimated curve of Ω and the observed data, plus two curves with lower transmission rates: $\beta_y = 0.59$ and $\beta_o = 0.68$ (both in $days^{-1}$), with $R_0 = 6.99$ (partials $R_{0y} = 5.84$ and $R_{0o} = 1.16$); and $\beta_y = 0.43$ and $\beta_o = 0.50$ (both in $days^{-1}$), with $R_0 = 5.09$ (partials $R_{0y} = 4.26$ and $R_{0o} = 0.84$). Fig C.1(b) shows the extended curves of Ω, from Eq (10), which approach asymptotes (or plateaus) indicating the end of the first wave of the epidemic. For $R_0 = 9.24$, 6.99, and 5.09, the curves Ω reach values with little difference on September 13, respectively, $946,400$, $945,700$, and $941,500$. For $R_0 = 9.24$, the curves for young (Ω_y) and elder (Ω_o) persons approach on September 13 values, respectively, $605,300$ and $341,100$.

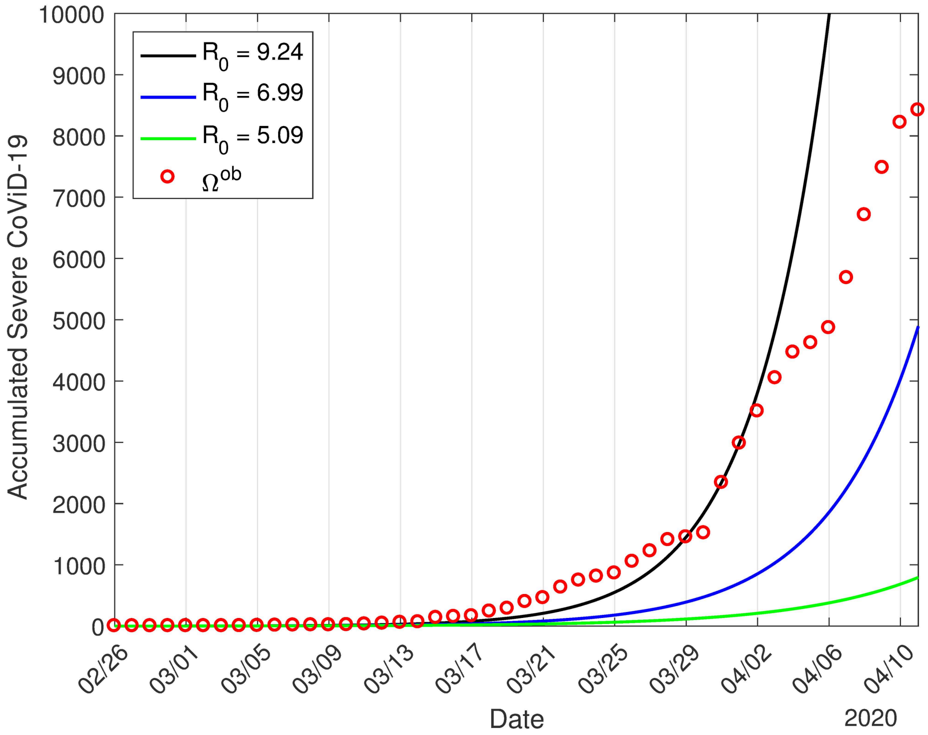

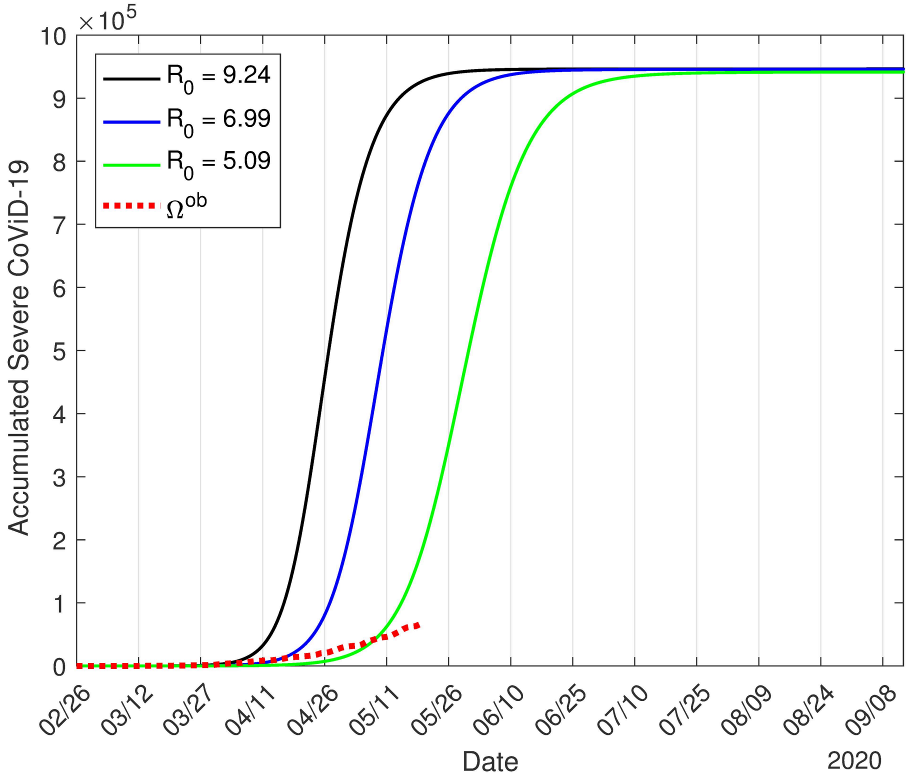

Fig C.1. The estimated curve of the accumulated number of severe covid-19 cases Ω in natural epidemic and observed data in São Paulo State, plus two curves with lower transmission rates: $\beta_y = 0.59$ and $\beta_o = 0.68$ ($days^{-1}$), with $R_0 = 6.99$, and $\beta_y = 0.43$ and $\beta_o = 0.50$ ($days^{-1}$), with $R_0 = 5.09$ (a), and extended curves of Ω (b).

We stress the fact that, if the observed data are fitted without caution about interventions, that is, using all data indistinctly, someone could estimate the basic reproduction number to be $R_0 = 5.09$ or less (notice that near the horizontal axis of Fig C.1(b), the observed data do not approach the curve of $R_0 = 5.09$, relatively lower R_0).

It is accepted that droplets transmit SARS-CoV-2 [24]. For instance, for the rubella infection, using seroprevalence data [25] and dealing with the SEIR model in the steady-state, the estimation was R_0=6.71 [10]. That estimation was done before the mass vaccination against rubella infection, which was why the estimation was the basic reproduction number. However, the rubella virus was circulating probably not in a steady-state, then the estimate would not be R_0, but the effective reproduction number R_{ef}, given by Eq (A.18), and the actual value of R_0 must be higher than 6.71. Moreover, Caieiras City in São Paulo State had $30,000$ inhabitants with a demographic density of $264/km^2$ in 1990, indicating that $R_0 = 6.71$ is under-estimation for rubella transmission in São Paulo State. As we pointed out in Materials and Methods, we have only two moments during the epidemic when R_0 can be estimated from data: At the beginning without any kind of constraints (during the natural epidemic), and when the steady-state is reached, which in general occurs after a long time [10].

In Appendix D.2, the incubation period $\sigma^{-1} = 5.8$ *days* corresponds to the mean value between 5.2 and 6.4. This range can be written as $\sigma^{-1} \pm \Delta\sigma^{-1}$, where $\Delta\sigma^{-1} = 0.6$ *days*. We can include the error in other parameters, writing as $u_i \pm \Delta u_i$. Taking into account the mean values (those given in Table 2) and the corresponding errors, we can perform the sensitivity analysis of R_0 using Eqs (A.23) and (A.24) presented in Appendix A.3. Table C.1 shows the contribution of each parameter to the variation ΔR_{0j} and error $\varrho_{R_{0j}}$. We choose arbitrarily equal relative error $\Delta u_i/u_i = 25\%$ for all parameters. The variation and error for R_0 are $\Delta R_0 = \Delta R_{0y} + \Delta R_{0o}$ and $\varrho_{R_0} = \sqrt{\varrho^2_{R_{0j}} + \varrho^2_{R_{0j}}}$. The constant fraction of susceptible persons are $s^0_y = 0.847$ and $s^0_o = 0.153$.

Table C.1. The sensitivity analysis of R_{0y} (young subpopulation), where u_{ji}, and $\langle u_{ji} \rangle$, and Δu_{ji} represent the parameter, its mean value and error, and $\varrho_{u_{ji}} = H_{ji}\Delta u_{ji}$, with $j = y, o$ and $i = 1, \cdots, 11$. The relative error is $\Delta u_{ji}/u_{ji} = 25\%$ for all parameters. The Sum stands for the approximated ΔR_{0j} and $\varrho_{R_{0j}}$. The rank in the contribution is given between parentheses. The error $\varrho_{R_{0j}} \approx \sqrt{\sum_{i=1}^{11} \varrho^2_{u_{ji}}}$ is 2.43 and 0.18 for young and elder subpopulations. Units are omitted.

u_{ji}	$\langle u_{yi} \rangle$	$\varrho_{u_{yi}}$	$\varrho^2_{u_{yi}}$	$\langle u_{oi} \rangle$	$\varrho_{u_{oi}}$	$\varrho^2_{u_{oi}}$
σ_j	1/5.8	0.000388	1.5×10^{-7} (11)	1/5.8	0.0000924	8.54×10^{-9} (11)
γ_j	1/12	−1.56919	2.462357 (2)	1/14	2.462357	0.1639772 (2)
γ_{1j}	1/4	−0.130812	0.017112 (10)	1/4	−0.0289423	0.0008377 (6)
γ_{3j}	1/13	−0.212446	0.045133 (7)	1/16	−0.0219749	0.0004829 (9)
β_{1j}	0.78	1.569850	2.464429 (1)	0.9	0.4051420	0.1641400 (1)
β_{2j}	0.78	0.130858	0.017124 (9)	0.9	0.0289488	0.0008380 (5)
β_{3j}	0.78	0.212542	0.045174 (5)	0.9	0.0219872	0.0004834 (7)
χ_j	0.98	−0.832952	0.693809 (3)	0.95	−0.0879245	0.0077307 (4)
p_j	0.8	0.281248	0.281248 (4)	0.8	0.2245360	0.0504164 (3)
m_j	0.92	0.195543	0.038237 (8)	0.75	0.0173596	0.0003014 (10)
z_j	0.5	0.212542	0.045174 (6)	0.2	0.0219872	0.0004834 (8)
	Sum	−0.142429	5.907650	Sum	0.1762705	0.0310713

From Table C.1, we can write $R_{0y} = 7.73 \pm 2.43$ and $R_{0o} = 1.51 \pm 0.18$ for, respectively, young and elder subpopulations. Hence, the basic reproduction number can be written as $R_0 = 9.24 \pm 2.61$, on the range $[6.63, 11.85]$ considering 25% error in all parameters.

C.1.2 Epidemic with isolation – Evaluating the proportion of the population in isolation

Isolation was introduced on March 24 (t_1), and we evaluate the proportion in isolation, taking into account the confirmed cases of CoViD-19 until April 12 (t_{20}) and using Eq (D.1).

To evaluate the proportion of the population in isolation, we fix the transmission rates $\beta_y = 0.78$ and $\beta_o = 0.90$ (both in $days^{-1}$), and vary $k =$ 0, 0.4, 0.53, 0.6, 0.7, and 0.8, where $k_{mean} = 0.53$ is the average proportion of persons in isolation from March 24 to May 3 (see B.2). We observe that $k = 0.4$ and 0.6 fit part of the observed data, while $k = 0.7$ does not. Hence, we chose $k = k_{mean} = 0.53$ as the value that explains xSão Paulo State's isolation. Fig C.2 shows the curves of Ω for different proportions in isolation in São Paulo State and the observed data (a) and the extended curves of Ω (b).

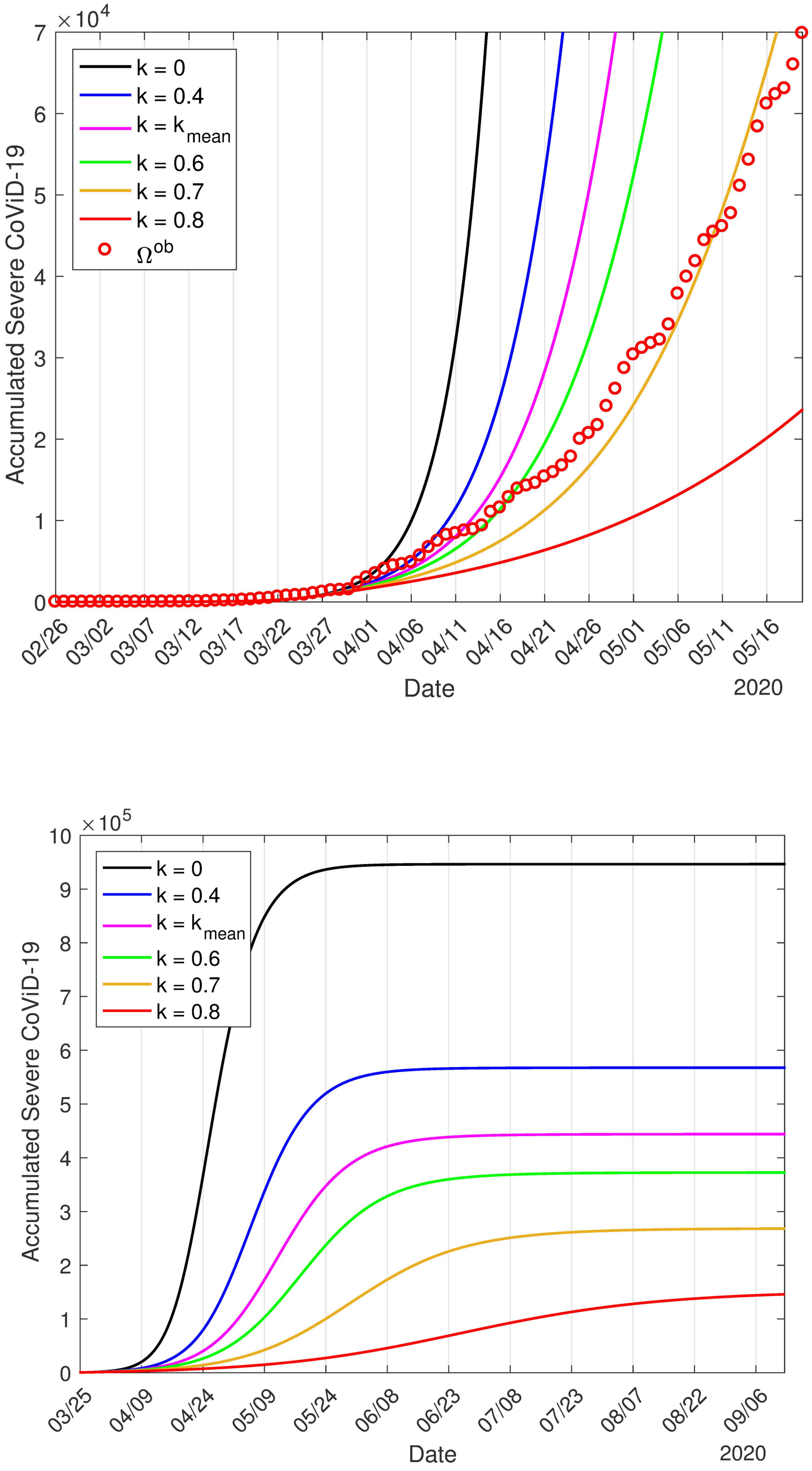

Fig C.2. The curves of Ω for the proportions in isolation in São Paulo State $k = 0$, 0.4, 0.53, 0.6, 0.7, and 0.8, and observed data (a), and the extended curves of Ω (b).

From Fig C.2(b), the curves Ω approach plateau, and the values on September 13 for $k = 0$, 0.4, 0.53, 0.6, 0.7, and 0.8 are, respectively, 964,600, 567,600 (60%), 444,000 (47%), 372,500 (40%), 268,200 (28%), and 146,000 (15%). The percentage between parentheses is the ratio $\Omega(k)/\Omega(0)$. For $k = k_{mean}$, the values for Ω_y and Ω_o are, respectively, 283,600 and 160,400.

As we have pointed out in the description of the data (Appendix B.1), the observed proportion in isolation delayed by approximately 9 days indeed affected the daily incidence of CoViD-19. Moreover, the chosen proportion k_{mean} is the average proportion of São Paulo State's isolation. The number of accumulated cases Ω decreases as k increases, showing that the population's isolation reduces the transmission of CoViD-19, flattening the epidemic curve. This decrease can also be assessed by Eq (A.20), which is the reduction in the effective reproduction number by isolation R_r.

When isolation is implemented during the epidemic, those who are harboring the virus can be found in the isolated population. At the time of the beginning of isolation in São Paulo State on March 24, we have

$$\begin{aligned} &\text{young}\left\{\begin{array}{llll} S_y = 17.8\text{million}, & Q_y = 19.9\text{million}, & E_y = 45,420, & A_y = 19,250, \\ D_{1y} = 3,200, & Q_{2y} = 2,395, & D_{2y} = 231 & \end{array}\right. \\ &\text{elder}\left\{\begin{array}{llll} S_o = 3.2\text{million}, & Q_o = 3.59\text{million}, & E_o = 9,434, & A_o = 4,153, \\ D_{1o} = 600, & Q_{2o} = 557, & D_{2o} = 166, & \end{array}\right. \end{aligned} \tag{C.1}$$

with $I = 10,032$. When 53% ($k = 0.53$) of the infectious persons in each class is transferred to the isolated classes, the total number of isolated persons harboring virus 43,490 can trigger a new epidemic in the population in isolation. However, if the transmission rate is low in the isolated population [23], the number of severe CoViD-19 cases is around 1% compared with the peak 67,140 shown below. Hence, we assume that SARS-CoV-2 is not transmitting in the isolated population, and we do not evaluate ω.

C.1.3 Epidemic with isolation and protective measures – Evaluating the reduction in the transmission rates

On April 13, 20 days after the beginning of isolation, we observe the first point leaving the curve completely. This new trend can not be explained by an increased proportion of isolation (see Fig B.2). To take into account this new tendency of data, in [6] we hypothesized that the using of face mask (protection of mouth and nose), protection of eyes, constant hygiene (washing hands with alcohol and gel), and social distancing might decrease the transmission of infection [26] and [27]. Different from isolation, these protective measures reduce the transmission rates, as can be seen in Eq (A.1).

We consider that on April 4, 9 days before the observed points leaving the estimated curve of the epidemic with isolation consistently, the protective measures were adopted by the population, which reduced the transmission rates from β_y and β_o to $\beta'_y = \varepsilon\beta_y$ and $\beta'_o = \varepsilon\beta_o$. We fix the transmission rates $\beta_y = 0.78$ and $\beta_o = 0.90$ (both in $days^{-1}$), and the proportion in isolation $k = 0.53$ to evaluate the protective factor ε taking into account data from April 4 (t_1) to May 7 (t_{34}), and using Eq (D.1).

We vary $\varepsilon = 0.8$, 0.7, 0.6, 0.5, and 0.4, and the value $\varepsilon = 0.5$ is chosen to represent the protective measures adopted in São Paulo State. Fig C.3 shows the curves of Ω for $\varepsilon = 1$, 0.8, 0.7, 0.6, 0.5, and 0.4 plus natural epidemic ($k = 0$) and the observed data (a), and the extended curves of Ω for $\varepsilon = 1$, 0.7, 0.6, 0.5, and 0.4 (b). The curves Ω approach plateau, and on November 2, the values for $\varepsilon = 1$, 0.7, 0.6, 0.5 and 0.4 are, respectively, 444,000, 427,900 (97%), 413,500 (93%), 386,600 (87%), and 331,200 (75%). The percentage between parentheses is the ratio $\Omega(\varepsilon)/\Omega(0)$. For $\varepsilon = 0.5$, the values for Ω_y and Ω_o are, respectively, 243,800 and 142,800, which are decreased by 86% and 89% in comparison with isolation alone as intervention.

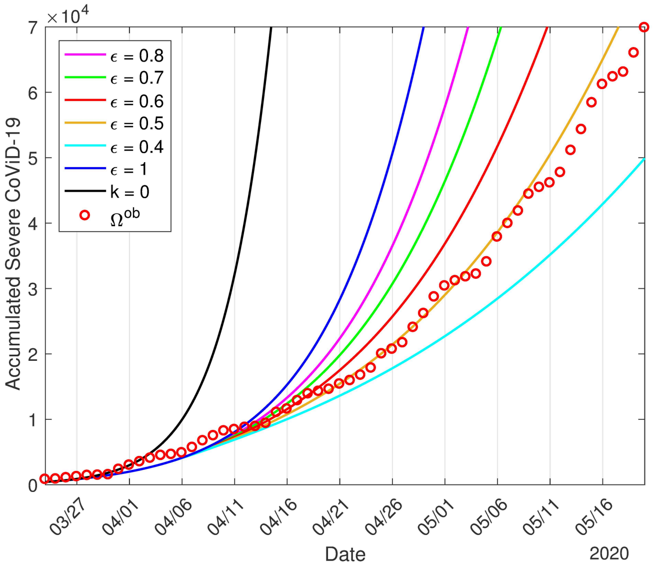

×10^4
ϵ = 0.8
ϵ = 0.7
ϵ = 0.6
ϵ = 0.5
ϵ = 0.4
ϵ = 1
k = 0
Ω^ob
Accumulated Severe CoViD-19
03/27
04/01
04/06
04/11
04/16
04/21
04/26
05/01
05/06
05/11
05/16
Date
2020

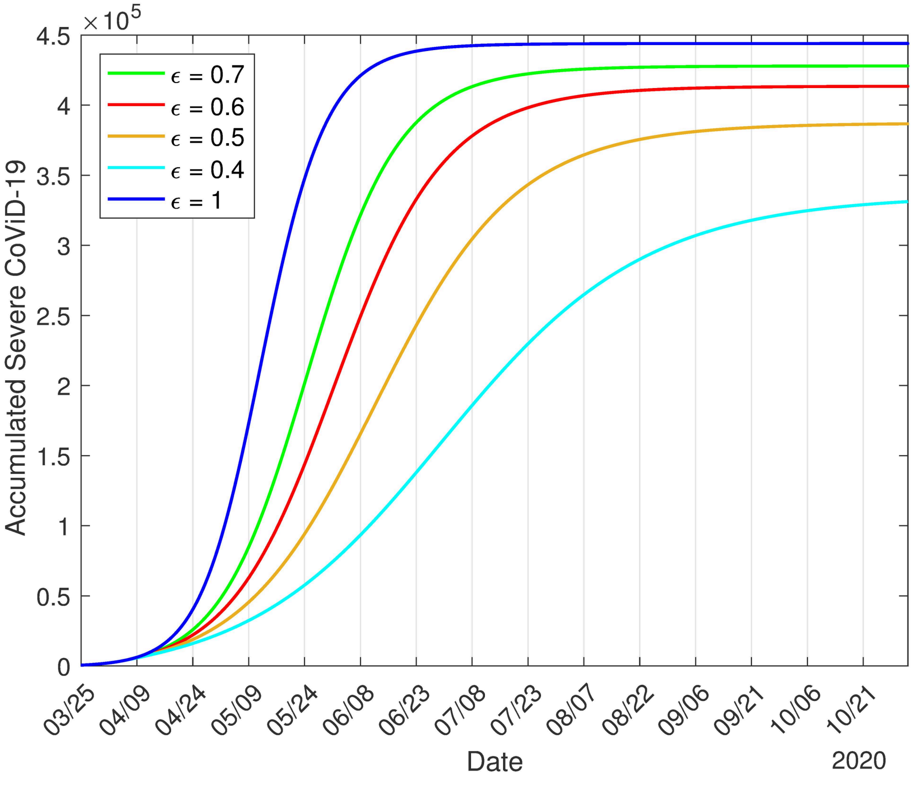

×10^5
ϵ = 0.7
ϵ = 0.6
ϵ = 0.5
ϵ = 0.4
ϵ = 1
Accumulated Severe CoViD-19
03/25
04/09
04/24
05/09
05/24
06/08
06/23
07/08
07/23
08/07
08/22
09/06
09/21
10/06
10/21
Date
2020

Fig C.3. The curves of Ω for the protective measures $\varepsilon = 1$, 0.8, 0.7, 0.6, 0.5 and 0.4, plus natural epidemic ($k = 0$) and the observed data (a), and the extended curves of Ω for $\varepsilon = 1$, 0.7, 0.6, 0.5 and 0.4 (b).

The number of accumulated cases Ω decreases as ε decreases ($1 - \varepsilon$ is the effectiveness of protective measures), showing that the populations's protective measures reduced the transmission of CoViD-19, flattening the epidemic curve. This decrease can also be assessed by Eq (A.21), which is the reduction in the effective reproduction number by protective measures R_p.

C.1.4 Evaluating the additional mortality rates

We estimate the additional mortality rates $\alpha_y = \Gamma\alpha_0$ and α_o taking into account confirmed deaths from March 16 (t_1) to May 20 (t_{66}) and using Eq (D.2). We fix the previously estimated transmission rates $\beta_y = 0.78$ and $\beta_o = 0.90$ (both in $days^{-1}$), the proportion in isolated population $k = 0.53$, and the protective factor $\varepsilon = 0.5$, to evaluate α_0. As we pointed out in Materials and Methods, we fix $\Delta = 15$ $days$ and let Γ =0.26 in São Paulo State (74% of deaths are occurring in elder persons with severe CoViD-19 [19]). The evaluated additional mortality rates are α_y =0.00185 and $\alpha_o = 0.0071$ (both in $days^{-1}$).

Fig C.4 shows the estimated curve of Π, from Eq (12), and the observed death data (a), and the extended curves of the number of CoViD-19 deaths for young Π_y, elder Π_o, and total $\Pi = \Pi_y + \Pi_o$ persons (b). The estimated curves Π_y, Π_o, and Π reach plateaus, and on November 2 the values are, respectively, 5,280 (2.2%), 18,500 (13%), and 23,780 (6.0%). The percentage between parentheses is the severe CoViD-19 case fatality rate Π/Ω, Ω being given in Fig C.3(b).

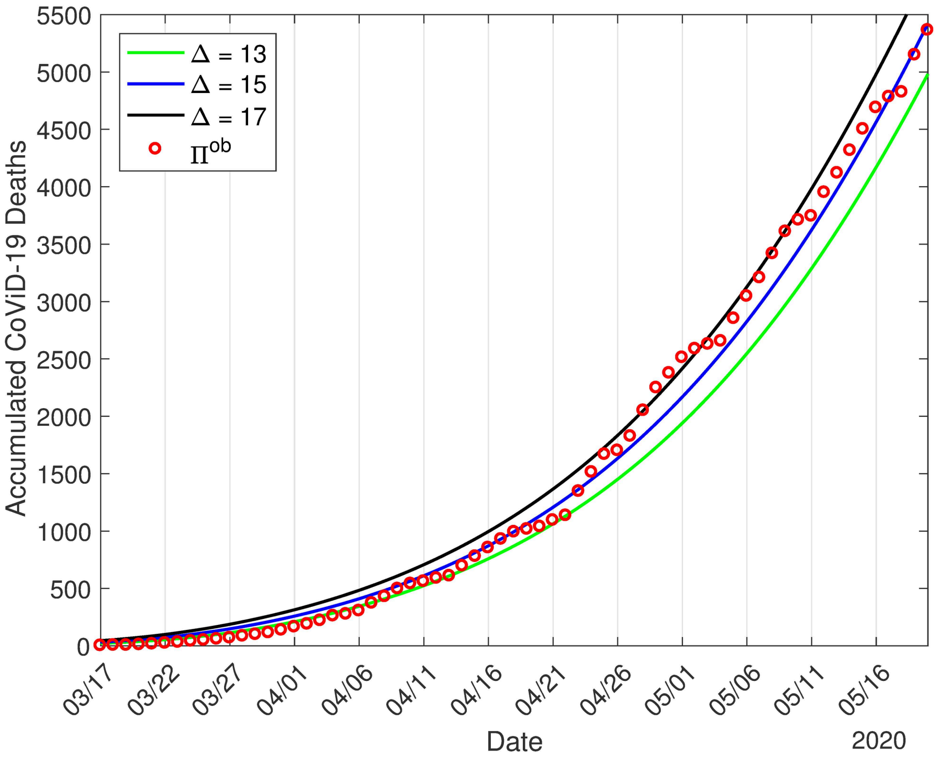

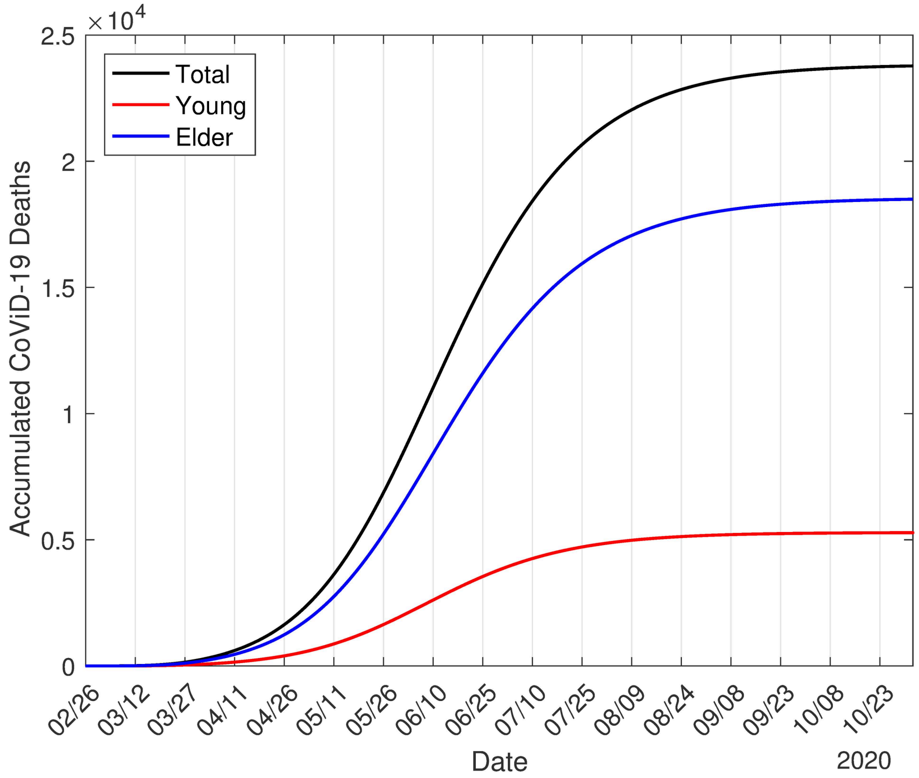

Fig C.4. The estimated curves of the accumulated deaths due to covid-19 Π for $\Delta = 13$, 15 and 17 *days*, and the observed data in São Paulo State (a), and the extended curves for young Π_y, elder Π_o, and total $\Pi = \Pi_y + \Pi_o$ persons for $\Delta = 15$ *days* (b).

At the end of the first wave of the epidemic, 78% of all deaths occurred in elder subpopulation. The number of the new cases of CoViD-19, from Eq (9), for young Φ_y, elder Φ_o, and total $\Phi = \Phi_y + \Phi_o$ persons are, respectively, 15.3 million, 2.86 million, and 18.16 million. The infection fatality rate (Π/Φ) in young, elder, and all persons are, respectively, 0.031%, 0.65%, and 0.13%.

C.2 Spain

Using data from January 31 to May 20 collected from Spain, we evaluate the transmission (β_y and β_o) rates, the proportion in the isolated populations (k), reduction in the transmission rates by protective measures adopted by population (ε) and social distancing (ω), and the additional mortality rates (α_y and α_o)

C.2.1 Natural epidemic – Evaluating the transmission rates

We evaluate the transmission rates during the natural epidemic taking into account the confirmed cases from January 31 (t_1) to March 21 (t_{51}), and using Eq (D.1). The evaluated values are $\beta_y = 0.67$ and $\beta_o = 0.74$ (both in $days^{-1}$), where $\psi = 1.1$, resulting in the basic reproduction number $R_0 = 8.0$ (partials $R_{0y} = 5.81$ and $R_{0o} = 2.19$), according to Eq (A.19). Fig C.5 shows the estimated curve of Ω and the observed data

(a), and the extended curves for young Ω_y, elder Ω_o, and total Ω persons (b). On June 30, the accumulated cases for young Ω_y elder Ω_o persons approach plateaus, assuming, respectively, $564,000$, $611,000$, and $1,175,000$.

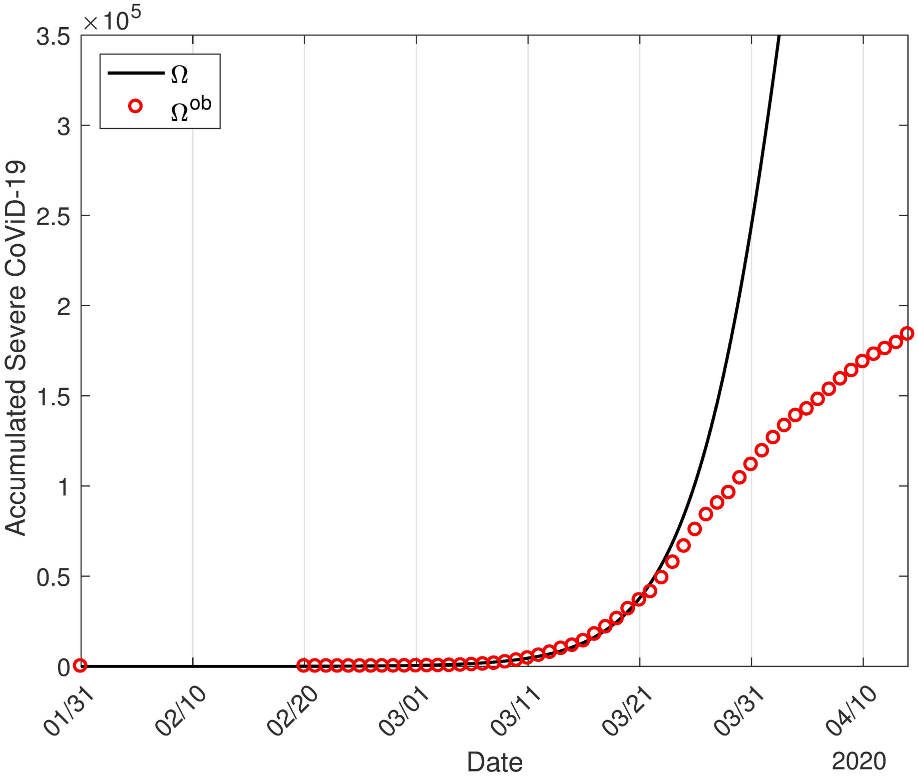

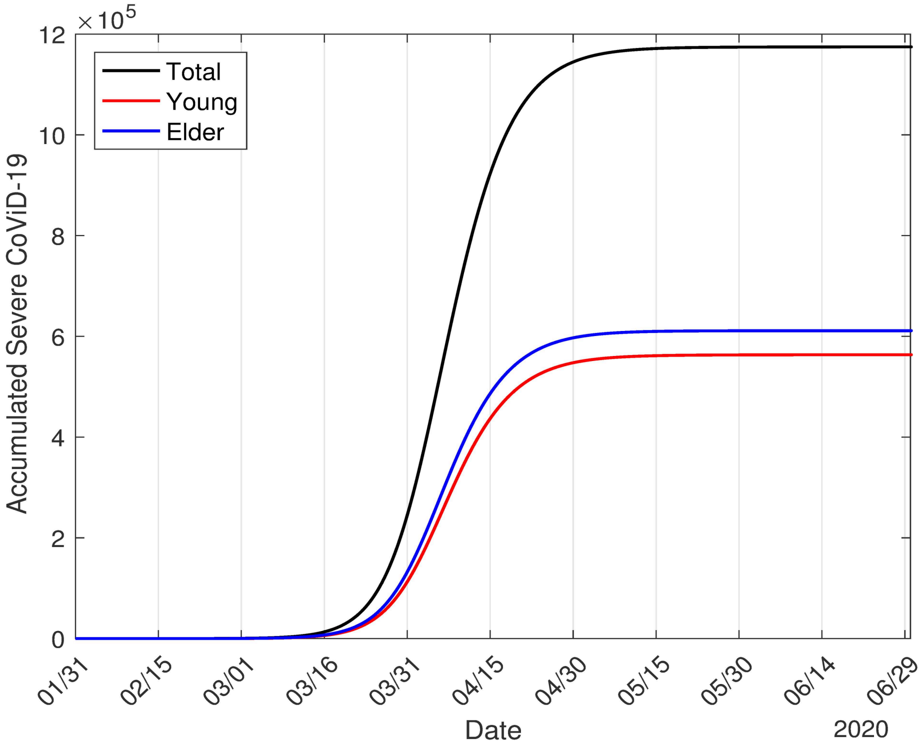

Fig C.5. The estimated curve of the severe covid-19 cases Ω for the natural epidemic and observed data in Spain (a), and the extended curves (b).

The estimated basic reproduction number R_0 in Spain is lower than in São Paulo State. The inhabitants in Spain is 6% higher, but the demographic density is 48% lower than those in São Paulo State. This decrease by almost half in the demographic density may explain the 13% lower in the basic reproduction number. The elder population in Spain is 40% higher than São Paulo State, and the evaluated ψ is 4.4% lower than that estimated for São Paulo State.

C.2.2 Transition epidemic – Evaluating the reduction in the transmission rates in circulating and lockdown populations

On March 16, the lockdown was implemented in Spain. But the data described in Materials and Methods showed a period (from March 22 to 28) of transition from natural epidemic to lockdown, effectively reducing the epidemic. Hence, we must consider CoViD-19 transmission in circulating and lockdown populations.

Initially, following the procedure adopted in São Paulo State, we vary the proportion of the population in lockdown, $k = 0.5$, 0.6, 0.7, 0.8, and 0.9, and evaluate the epidemic in the circulating population. Notice that the circulating population's epidemic must be lower than the observed cases of severe CoViD-19 if the infection is also happenig in the lockdown population. Fig C.6(a) below shows that, for the proportions 0.8 and 0.9 in lockdown, the occurrence of infections in the circulating population is lower than the observed data in Spain, but $k = 0.7$ shows the number of cases provided by the model following sigmoid-shape and increasing beyond the observed data.

Hence, we chose $k = 0.9$ to represent the lockdown in Spain, instead of 80%, and evaluate the epidemic in circulating and lockdown populations by fixing the transmission rates $\beta_y = 0.67$ and $\beta_o = 0.74$ (both in $days^{-1}$). We consider that during the transition of the epidemic, the populations in circulation ($k = 0.1$) and lockdown ($k = 0.9$) did not adopt any protective measure ($\varepsilon = 1$), but the people in lockdown has restricted contact ($\omega > 1$). Hence, taking into account the confirmed cases from March 22 (t_1) to 28 (t_7) and using Eq (D.1), the evaluated decreasing factor in the population in lockdown is $\omega = 1.5$, resulting in $\beta'_y = 0.45$ and $\beta'_o = 0.49$ (both in $days^{-1}$). Fig C.6 shows the curves of Ω for $k = 0.5$, 0.6, 0.7, 0.8, and 0.9 and the observed data (a), and the curves of Ω for the natural epidemic and epidemic in the transition phase (b).

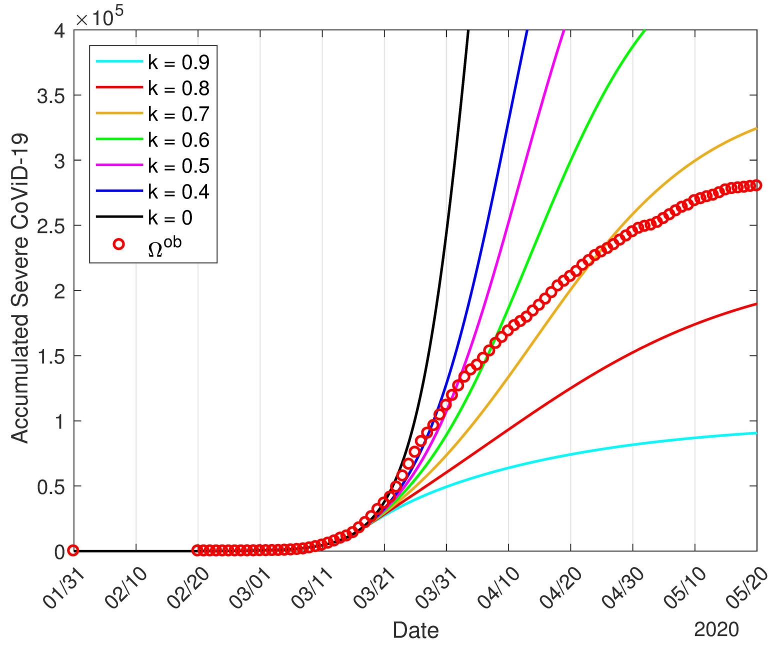

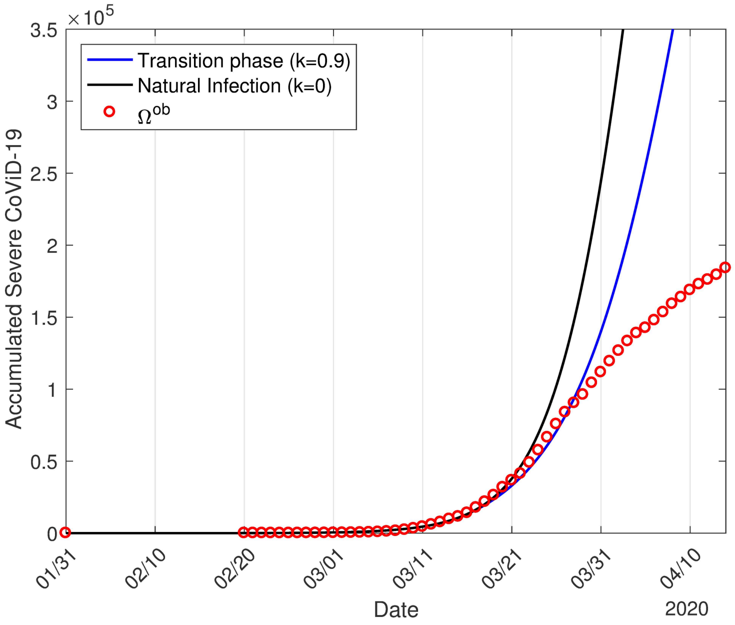

Fig C.6. The curves of Ω for the proportions in isolation in Spain $k = 0$, 0.4, 0.5, 0.6, 0.7, 0.8, and 0.9, and observed data (a), and the estimated curves of Ω for natural and transition epidemic considering $k = 0.9$ in population in lockdown (b).

When a lockdown is implemented during the epidemic, those who are harboring the virus can be found in the isolated population. At the time of the beginning of lockdown

on March 16, we have

$$\text{young}\begin{cases} S_y = 3.39\text{million}, & Q_y = 31.7\text{million}, & E_y = 69,713, & A_y = 32,639, \\ D_{1y} = 5,221, & Q_{2y} = 4,332, & D_{2y} = 3,520 \end{cases}$$
$$\text{elder}\begin{cases} S_o = 1.17\text{million}, & Q_o = 11.0\text{million}, & E_o = 26,601, & A_o = 12,988, \\ D_{1o} = 1,994, & Q_{2o} = 1,527, & D_{2o} = 4,602, \end{cases} \tag{C.2}$$

with $I = 188,430$. When 90% ($k = 0.9$) of the infectious persons in each class is transferred to the isolated classes, the total number of isolated persons harboring virus $134,240$ can trigger a new epidemic in the population in lockdown with an elevated number of susceptible persons. When a new epidemic begins in the population in lockdown, the classes Q_y and Q_o become S_y and S_o.

C.2.3 Epidemic with lockdown – Evaluating the proportion of the population in lockdown and reduction in the transmission rates

Lockdown was implemented on March 16, but the actual effects began after the transition period. We fix the previously estimated transmission rates $\beta_y = 0.67$ and $\beta_o = 0.74$ (both in $days^{-1}$) in the natural epidemic, and the reduction in the transmission rate $\omega = 1.5$ in the population in lockdown ($k = 0.9$) in the epidemic in the transition period. The parameters ε and ω corresponding to the third period of the epidemic are evaluated, taking into account the confirmed cases from March 24 (t_1) to May 20 (t_{57}), and using Eq (D.1).

We evaluate the changes in the protective measures (ε) in the circulating population and decreased transmission rates (ω) in the lockdown population. In the circulating population, the evaluated protective factor is $\varepsilon = 0.5$, resulting in $\beta'_y = 0.34$ and $\beta'_o = 0.391$ (both in $days^{-1}$). In contast, in the population in lockdown, the evaluated decreasing factor is $\omega = 11.5$, resulting in $\beta'_y = 0.059$ and $\beta'_o = 0.068$ (both in $days^{-1}$). Fig C.7 shows the estimated curves of Ω and the observed data (a), and the illustration of the curves of D_2 occurring in circulating (continuous) and lockdown (dashed) populations (b). The estimated curves Ω_y, Ω_o, and $\Omega = \Omega_y + \Omega_o$ approach plateaus, and on October 7, the values are, respectively, $147,100$, $173,100$, and $320,200$.

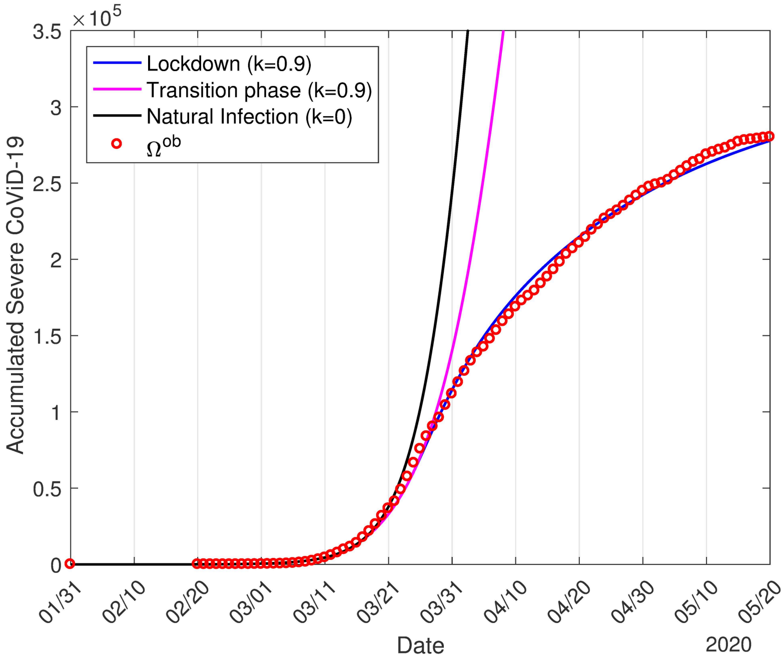

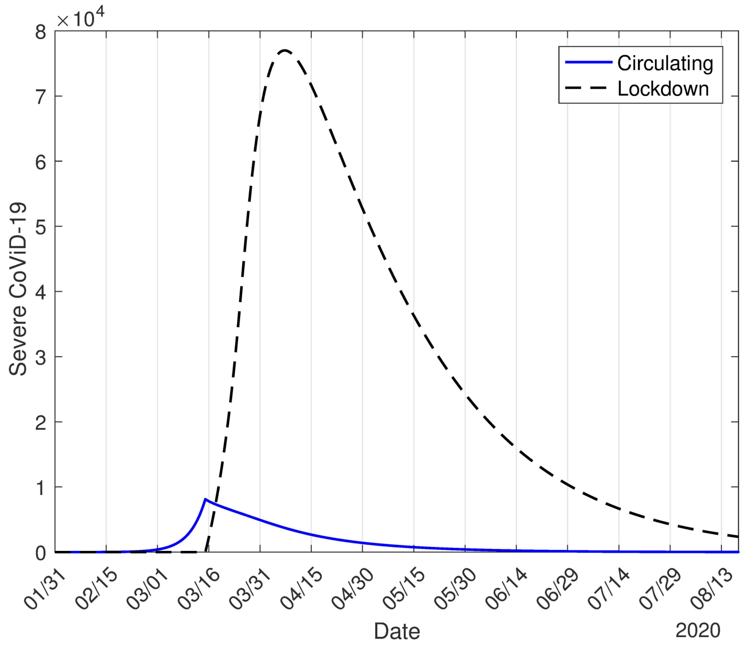

Fig C.7. The estimated curves of Ω for natural epidemic, epidemic in transition phase, and epidemic in lockdown and observed data in Spain (a), and severe covid-19 cases in circulating and locked-down population (b). The flattened curve in (a) is the sum of two curves in (b).

For $k = 0.8$, better evaluations in ε and ω were obtained considering the effectiveness of lockdown appearing on March 25, exactly 9 days after March 16. The estimated protective factor in the circulating population is $\varepsilon = 0.45$, resulting in $\beta'_y = 0.306$ and $\beta'_o = 0.352$ (both in $days^{-1}$), and for the lockdown population, the decreasing factor in the transmission rates is $\omega = 12.3$, resulting in $\beta'_y = 0.055$ and $\beta'_o = 0.064$ (both in $days^{-1}$).

C.2.4 Evaluating the additional mortality rates

Taking into account confirmed deaths from March 8 (t_1) to May 20 (t_{73}) and using Eq (D.2), we evaluate the additional mortality rates $\alpha_y = \Gamma\alpha_0$ and α_o. To assess the mortality rates, we fix the previously estimated transmission rates $\beta_y = 0.67$ and $\beta_o = 0.74$ (both in $days^{-1}$) in the natural epidemic, the reduction in the transmission rate $\omega = 1.5$ in the population in lockdown ($k = 0.9$) in the epidemic during the transition, and when lockdown effectively affects epidemic, protection factor $\varepsilon = 0.5$ in

the people in circulation and reduction in the transmission $\omega = 11.5$ in the lockdown population.

We consider Δ=15 *days* and let Γ =0.05 in Spain (95.5% of deaths are occurring in the elder persons with severe CoViD-19 [9]), and the evaluated additional mortality rates are $\alpha_y = 0.00273$ and $\alpha_o = 0.0105$ (both in $days^{-1}$). Fig C.8 shows the estimated curve of Π, from Eq (12), and the observed death data (a), and the extended curves of the number of CoViD-19 deaths for young Π_y, elder Π_o, and total $\Pi = \Pi_y + \Pi_o$ persons (b). The estimated curves Π_y, Π_o, and Π approach plateaus, and on October 7, the values are, respectively, 920 (0.6%), 31, 230 (18%), and 32, 150 (10%). The percentage between parentheses is the severe CoViD-19 case fatality rate Π/Ω.

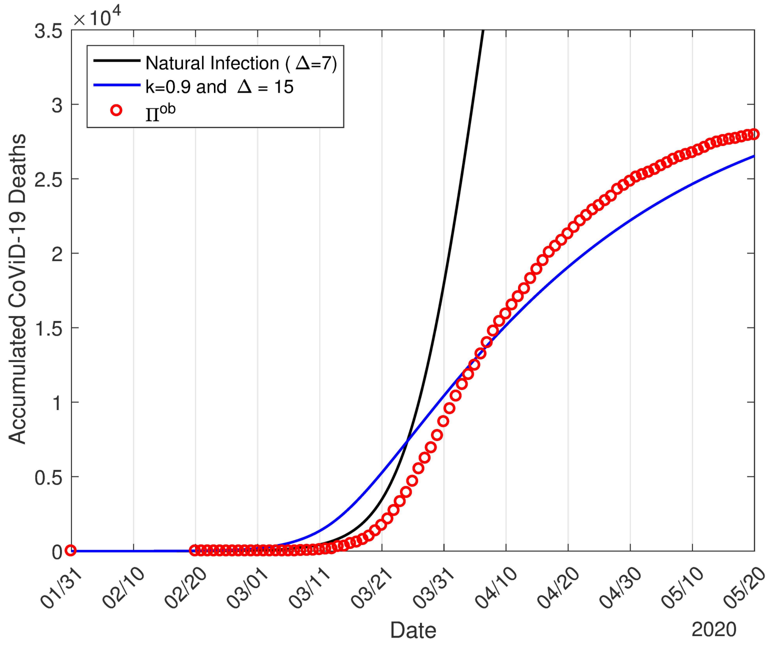

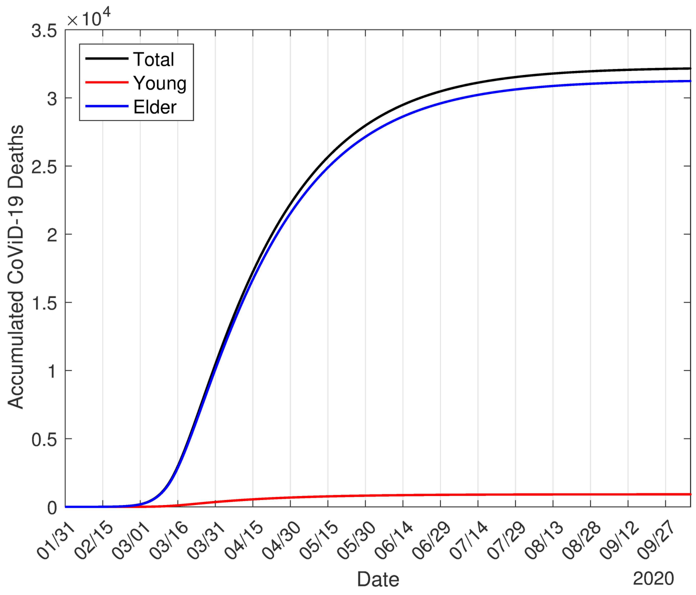

Fig C.8. The estimated curve of accumulated deaths due to covid-19 Π and the observed data in Spain (a), and the extended curves for young Π_y, elder Π_o, and total $\Pi = \Pi_y + \Pi_o$ persons (b).

At the end of the first wave of the epidemic, 97% of all deaths occurred in elder subpopulation. The number of the new cases of CoViD-19, from Eq (9), for young Φ_y, elder Φ_o, and total $\Phi = \Phi_y + \Phi_o$ persons are, respectively, 9.21 million, 3.47 million, and 12.68 million. The infection fatality rate (Π/Φ) in young, elder, and all persons are, respectively, 0.01%, 0.9%, and 0.25%.

D Estimation method and the values for the model parameters

The values assigned in Table 2 are obtained from the estimation of CoViD-19 cases and deaths, or they are calculated.

D.1 Model parameters evaluated from the data

The model parameters can be fitted applying the non-linear least square estimation method (see [28]), that is,

$$\min \sum_{i=1}^{n} \varsigma_i \left[Z(t_i) - Z^{ob}(t_i) \right]^2,$$

where min stands for the minimum value, n is the number of observations, t_i is i-th observation time, Z stands for Ω given by Eq (10), or Π given by Eq (12), and Z^{ob} stands for the observed number of severe CoViD-19 cases Ω^{ob} or the number of died persons Π^{ob}. We let $\varsigma_i = 1$ for the weights. However, this estimation method of parameters is hugely complex and not appropriate (see [29]) when there are observed data in only one variable of the dynamic system. For this reason, we evaluate the sum of squared differences by varying model parameters. This simplified method of parameter evaluation does not provide uncertainties associated with the parameters.

To evaluate the transmission rates in the natural epidemic (there are not any interventions at the beginning of the epidemic, then $\varepsilon = \omega = 1$), we assume that all rates in young persons are equal, as well as in elder persons, letting

$$\beta_y = \beta_{1y} = \beta_{2y} = \beta_{3y} = \beta_{1o} = \beta_{2o} = \beta_{3o}, \qquad \text{and} \qquad \beta_o = \psi \beta_y,$$

and the forces of infection are $\lambda_y = (A_y + D_{1y} + z_y Q_{2y} + A_o + D_{1o} + z_o Q_{2o}) \beta_y / N$ and $\lambda_o = \psi \lambda_y$. The reason to include a factor ψ is the reduced capacity of a defense

mechanism by elder persons (physical barrier, innate and adaptive immune responses, etc.). Another fact is the expression of angiotensin-converting enzyme 2 in the nasal epithelium, the receptor that SARS-CoV-2 uses for host entry, which increases with age [30]. The force of infection takes into account all virus released by infectious individuals (A_y, D_{1y}, Q_{2y}, A_o, D_{1o} and Q_{2o}), the rate of encounter with susceptible persons (many factors, especially demographic density and closeness), and the capacity to infect them (see [31] and [32]). Additionally, the amount inhaled by susceptible persons can be determinant in the chance of infection, and the prognosis of CoViD-19 [33]. We also assume that the proportions in the isolated young and elder subpopulations are equal, that is, $k = k_y = k_o$.

Currently, there is not a sufficient number of kits to detect infection by the new coronavirus. For this reason, test to confirm infection by this virus is done only in hospitalized persons; and in persons who died manifesting symptoms of CoViD-19. Hence, we have only data of accumulated severe CoViD-19 cases ($\Omega = \Omega_y + \Omega_o$) and those who died ($\Pi = \Pi_y + \Pi_o$).

To evaluate the parameters β_y and $\beta_o = \psi\beta_y$, the proportions in the isolated population k, and reduction in the transmission rates due to protective measures adopted by population ε and decreased contact ω, we calculate

$$\sum_{i=1}^{n} \left[\Omega\left(t_i\right) - \Omega^{ob}\left(t_i\right)\right]^2 \tag{D.1}$$

by varying the parameters, and we choose the lower sum of the squared distances between the curve and data. The accumulated CoViD-19 cases Ω is given by Eq (10).

To estimate the mortality rates α_y and α_o, the let $\alpha_y = \Gamma\alpha_o$, where Γ is provided by the ratio of deaths occurring in young and elder subpopulations. We fix the previously estimated parameters β_y and β_o, k, ε, and ω, and evaluate

$$\sum_{i=1}^{n} \left[\Pi\left(t_i + \Delta\right) - \Pi^{ob}\left(t_i\right)\right]^2, \tag{D.2}$$

by varying the parameter α_o. The accumulated deaths due to CoViD-19 Π is given by Eq (12), with $\Pi(0) = 0$. Notice that the time of registration t_i of deaths must be related to the deaths of new cases Δ times ago, that is, $D_2(t_i - \Delta)$. We use $\Delta = 15$ *days* obtained by analyzing the data from São Paulo State.

D.2 Assignment of values for the model parameters

Some values for model parameters are found in the literature, and other parameters are calculated.

For incubation period, we use mean value between 5.2 [34] and 6.4 [35], that is, $\sigma = \sigma_y = \sigma_o = 1/5.8\ days^{-1}$. We use for the infectious rates of pre-diseased persons, $\gamma_1 = \gamma_{1y} = \gamma_{1o} = 1/4\ days^{-1}$ [36], which is indirectly confirmed by the delay observed in 9 days between low isolation and increase in CoViD-19 cases. It was observed approximately 2 weeks for the duration of mild disease, then we use $\gamma_o = 1/14$ and $\gamma_y = 1/12$ (both in $days^{-1}$), and acute disease lasts 2-6 weeks, then we use $\gamma_{2o} = 1/21$ and $\gamma_{2y} = 1/12$ (both in $days^{-1}$) [37].

Li *et al.* [38] observed that 86% of all infections were undocumented, and assuming that the ratio between asymptomatic and symptomatic young and elder persons are equal, we let $p = p_y = p_o = 4/5 = 0.8$. Additionally, Pan *et al.* [39] observed that one had clinical symptoms in a family of three, and the other two members were both asymptomatic. From São Paulo State, 76% of deaths due to CoViD-19 are 60 years old or more, then the ratio of death is $1:3$ to young persons [19]. However, the ratio may

be lower in severe CoViD-19 cases, and then we assume $2:3$ (in São Bernardo de Campo City, São Paulo State, the ratio of hospitalized young and elder persons is $2:3.3$). From the observation that 81% are mild and can recover at home [40, we consider a lowered ratio between mild and severe CoViD-19 among elder persons $3:1$, hence $m_o = 3/4 = 0.75$. To have a ratio of $2:3$ between young and elder in hospitalized persons, we must have approximately $10:1$ in the ratio between asymptomatic and symptomatic among young persons. Gathering the above information, we calculate approximately

$$c = \frac{1-b}{b} \times \frac{1}{4} \times \frac{2}{3},$$

where the term $(1-b)/b$ is the populational ratio between young and elder persons, $1/4$ is the proportion of severe CoViD-19 cases among elder persons, and $2/3$ is the ratio between hospitalized young and elder persons. Using $b = 0.153$, we have $c = 0.92$. Hence, the ratio asymptomatic:symptomatic of young persons is approximately $12:1$, which results in $m_y = 12/13 = 0.92$, for $p_y = p_o$.

The dynamic of the new coronavirus propagation without mass test and relaxation is obtained by evaluating the system of Eqs (1), (2), and (3) numerically using the 4th order Runge-Kutta method. Let us determine the initial conditions supplied to this system. In São Paulo State, the number of inhabitants is $N(0) = N_0 = 44.6$ million [41]. The value of parameter φ given in Table 1 was calculated by rewriting the Eq (A.9) as $\varphi = b\phi/(1-b)$, where b is the proportion of elder persons. Using $b = 0.153$ in São Paulo State [41], we obtained $\varphi = 6.7 \times 10^{-6}\, days^{-1}$, hence, $N_y(0) = N_{0y} = 37.8$ million ($s^0{}_y = N_{0y}/N_0 = 0.847$) and $N_o(0) = N_{0o} = 6.8$ million ($s_o{}^0 = N_{0o}/N_0 = 0.153$). Hence, the initial conditions for susceptible persons are $S_y(0) = N_{0y}$ and $S_o(0) = N_{0o}$ for S˜ao Paulo State. Following the same idea, the initial conditions for Spain are $S_y(0) = 35.17$ million and $S_o(0) = 12.23$ million.

The initial conditions for other variables are calculated based on Table 2. Using $p_y = p_o = 0.8$, the ratio asymptomatic:symptomatic is $4:1$ for young and elder persons; using $m_o = 0.75$, the ratio mild:severe CoViD-19 is $3:1$ for elder persons, and for young persons, the ratio is $12:1$ from $m_y = 0.92$. Hence, for elder subpopulation, if we assume that there is one person in D_{2o} (the first confirmed case), then there are 3 persons in Q_{2o}; the sum 4 is the number of persons in D_{1o}, implying that there are 16 in A_o, hence, the sum 20 is the number of persons in E_o. Notice that if there is 1 person in D_{2y}, there must be 12 persons in Q_{2y}. For young subpopulation, we assume that there is not any person in D_{2y}, but 6 persons in Q_{2y}, then the sum 6 is the number of persons in D_{1y}, implying that there are 24 in A_y, hence, the sum 30 is the number of persons in E_y. Finally, we suppose that no one is isolated or tested and immunized.

Therefore, the initial conditions supplied to the dynamic system (1), (2), and (3) are, for young and elder subpopulations,

$$\begin{cases} \text{young}: \begin{cases} S_y(0) = N_{0y}, \quad Q_y(0) = 0, \quad E_y(0) = 30, \\ A_y(0) = 24, \quad D_{1y}(0) = 6, \quad Q_{2y}(0) = 6 \quad D_{2y}(0) = 0, \end{cases} \\ \text{and} \\ \text{elder}: \begin{cases} S_o(0) = N_{0o}, \quad Q_o(0) = 0, \quad E_o(0) = 20, \\ A_o(0) = 16, \quad D_{1o}(0) = 4, \quad Q_{2o}(0) = 3 \quad D_{2o}(0) = 1, \end{cases} \end{cases} \tag{D.3}$$

plus $I(0) = 0$, where the initial simulation time $t = 0$ corresponds to the calendar time when the first case was confirmed (February 26 for São Paulo State, and January 31 for Spain).

Data in Supporting Information file

Captions

Table 1. Daily registered severe CoViD-19 cases and deaths from São Paulo State. The first date (0) corresponds to the calendar time February 26, and the last date (84), to the calendar time May 20. Available at https://www.saopaulo.sp.gov.br/coronavirus#numero-vacinacao.

Table 2. Daily registered proportion in isolation in São Paulo State. The first date (27) corresponds to the calendar time March 24 when partial quarantine was implemented, and the last date (84), to the calendar time May 20. Avaiable at https://www.saopaulo.sp.gov.br/coronavirus/isolamento.

Table 3. Daily registered severe CoViD-19 cases and deaths from Spain. The first date (0) corresponds to the calendar time January 31, and the last date (110), to the calendar time May 20. None cases and deaths were registered from date 1 to 19. Available at https://cnecovid.isciii.es/covid19/.

References

1. Anderson RM, May, RM. Infectious Diseases of Human. Dynamics and Control. Oxford, New York, Tokyo: Oxford University Press; 1991: 757 p.

2 Yang. HM, Ferreira WC. A populational model applied to HIV transmission considering protection and treatment. IMA J. Mat. appl. Med. Biol. 1999; 16: 237-259.

3. Diekmann O, Heesterbeek JAP, Roberts MG. The construction of next-generation matrices for compartmental epidemic models. J. R. Soc. Interface 2010; 7: 873-885.

4. Yang HM. The basic reproduction number obtained from Jacobian and next generation matrices – A case study of dengue transmission modelling. BioSystems 2014; 126: 52-75.

5. Yang HM, Greenhalgh D. Proof of conjecture in: The basic reproduction number obtained from Jacobian and next generation matrices – A case study of dengue transmission modelling. Appl. Math. Comput. 2015; 265: 103-107.

6. Yang HM, Lombardi Junior LP, Castro FFM, Campos AC. Mathematical model describing CoViD-19 in São Paulo State, Brazil – Evaluating isolation as control mechanism and forecasting epidemiological scenarios of release. Epidemiology and Infection 2020; 148: e155. doi: 10.1017/S0950268820001600.

7. Yang HM, *et al.* Fitting the incidence data from the City of Campinas, Brazil, based on dengue transmission modellings considering time-dependent entomological parameters. PlosOne 2016; 11(3): e0152186. https://doi.org/10.1371/journal.pone.

8. Yang HM. The transovarial transmission in the dynamics of dengue infection: Epidemiological implications and thresholds. Math. Biosc. 2017; 286: 1-15.

9. Yang HM. Are the beginning and ending phases of epidemics provided by next generation matrices? – Revisiting drug sensitive and resistant tuberculosis model. arXiv: 2006.06857 [Preprint]. 2020 [submitted June 11, 2020; accessed June 11, 2020]. Available from: http://arxiv.org/abs/2006.06857.

10. Yang HM. Modeling directly transmitted infections in a routinely vaccinated population – The force of infection described by Volterra integral equation. Applied Mathematics and Computation 2001; 122 (1): 27-58.

11. Yang HM. A mathematical model for malaria transmission considering global warming and local socio-economic conditions – The sensitivity analysis. Revista de Saúde Pública 2001; 35 (3): 224-231.

12. Yang HM. Modelling vaccination strategy against directly transmitted diseases using a series of pulses. Journal of Biological Systems 1998; 6 (2): 187-212.

13. Maidana NA, Yang HM. Describing the geographic spread of dengue disease by traveling waves, Mathematical Biosciences 2008; 215: 64-77.

14. Maidana NA, Yang HM. How do bird migrations propagate West Nile Virus. Math. Popul. Studies 2013; 20: 192-207.

15 Gray AJ, Greenhalgh D, Hu L, Mao X, Pan J. A stochastic differential equation SIS epidemic model. SIAM Journal on Applied Mathematics 2010; 71 (3): 876-902.

16. Freitas LFS. Vacinação de doenças infecciosas de transmissão direta : quantificando condições de controle considerando portadores. PhD Thesis, The State University at Campinas. 2018. Available from: http://www.repositorio.unicamp.br/handle/REPOSIP/331644.

17. Yang HM, Coutinho FAB, Massad E. Acquired immunity on a achistosomiasis transmission model – Fitting the data. Journal of Theoretical Biology 1997; 188: 495-506.

18. Ferreira CP, Yang HM, Esteva L. Assessing The Suitability of Sterile Insect Thecnique Applied to *Aedes aegypti*. Journal of Biological Systems 2008; 16: 565-577.

19. Casos em São Paulo; 2020 [accessed May 8, 2020]. Database [Internet]. Available from: https://www.saopaulo.sp.gov.br/coronavirus#numero-vacinacao.

20. Adesão ao isolamento social em SP; 2020 [accessed May 10, 2020]. Database [Internet]. Available from: https://www.saopaulo.sp.gov.br/coronavirus/isolamento.

21. Info.Gripe – Fiocruz; 2020 [accessed May 10, 2020]. Database [Internet]. Available from: http://info.gripe.fiocruz.br/.

22. COVID19; 2020 [accessed May 22, 2020]. Database Covid19 [Internet]. Available from: https://cnecovid.isciii.es/covid19/.

23. Yang HM, Lombardi Junior LP, Castro FFM, Campos AC. Evaluating reduction in CoViD-19 cases by isolation and protective measures in São Paulo State, Brazil, and scenarios of release. MedRxiv [Preprint]. 2020 medRxiv 20099309v2 [posted May 22, 2020; revised June 4, 2020; accessed June 5, 2020]: [55 p.]. Available from: https://www.medrxiv.org/content/10.1101/2020.05.19.20099309v2.

24. Zhu N, *et al.* A novel Coronavirus from patients with pneumonia in China, 2019. N. Eng. J. Med. 2020; 381: 1-7. doi: 10.1056/NEJMoa2001017.

25. Massad E, *et al.* Assessing the efficacy of a mixed vaccination strategy against rubella in São Paulo, Brazil. Intern. J. Epidem. 1995; 24 (4): 842-850.

26. Chu DK, *et al.* Physical distancing, face masks, and eye protection to prevent person-to-person transmission of SARS-CoV-2 and COVID-19: a systematic review and meta-analysis. The Lancet 2020; Published online June 1, 2020 https://doi.org/10.1016/S0140-6736(20)31142-9.

27. Howad J, *et al.* Face masks against CoViD-19: An evidence review. 2020; [posted April 12, 2020; accessed June 5, 2020]. doi:10.20944/preprints202004.0203.v1.

28. Raimundo SM, Yang HM, Bassanezi RC, Ferreira MAC. The attracting basins and the assessment of the transmission coefficients for HIV and *M. tuberculosis* infections among women inmates. Journal of Biological Systems 2002; 10 (1): 61-83.

29. Raimundo SM, Engel AB, Yang HM, Bassanezi RC. An approach to estimating the transmission coefficients for AIDS and for Tuberculosis using mathematical model. Systems Analysis Modelling Simulation 2003; 43 (4): 423-442.

30. Bunyavanich S, Do A, Vicencio A. Nasal gene expression of angiotensin-converting enzyme 2 in children and adults. JAMA 2020; Published Online: May 20, 2020. doi:10.1001/jama.2020.8707.

31. Yang HM. Directly transmitted infections modeling considering age-structured contact rate – Epidemiological analysis, Mathematical and Computer Modelling 1999: 29 (7): 11-30.

32. Yang HM. Directly transmitted infections modeling considering age-structured contact rate, Mathematical and Computer Modelling. 1999; 29 (8): 39-48.

33. Gomez MC, Yang HM. A simple mathematical model to describe antibody-dependent enhancement in heterologous secondary infection in dengue. Mathem. Med. Biol.: A Journal of the IMA 2029; 36: 411-438.

34. Qun L, *et.al*. Early transmission dynamics in Wuhan, China, of Novel Coronavirus-Infected Pneumonia. The New Engl. J. Medicine 2020; 182 (13): 1199-1207.

35. Backer JA, Klinkenberg D, Wallinga J. Incubation period of 2019 novel coronavirus (2019-nCoV) infections among travellers from Wuhan, China, 20-28 January 2020. Euro Surveill. 2020; 25 (5): pii=2000062. Available from: https://doi.org/10.2807/1560-7917.ES.2020.25.5.2000062.

36. Arons MM, *et al.* Presymptomatic SARS-CoV-2 infections and transmission in a skilled nursing facility. The New Engl. Jour. Medicine 2020; April 24, 2020. doi: 10.1056/NEJMoa2008457.

37. WHO. Report of the WHO-China Joint Mission on Coronavirus Disease 2019 (CoViD-19). 16-24 February 2020, 2020; pp 40.

38. Li RY, *et al.* Substantial undocumented infection facilitates the rapid dissemination of novel coronavirus (SARS-CoV2). Science 2020; 368(6490): 489-493. doi: 10.1126/science.abb3221.

39. Pan X, *et al.* Asymptomatic cases in a family cluster with SARS-CoV-2 infection. Lancet Infect Dis. 2020; 20 (4): 410–411. doi: 10.1016/S1473-3099(20)30114-6.

40. The Novel Coronavirus Pneumonia Emergency Response Epidemiology Team. The epidemiological characteristics of an outbreak of 2019 novel coronavirus diseases (CoViD-19) – China, 2020. CCDC Weekly 2020; 2 (8): 113-122.

41. SEADE – Fundação Sistema Estadual; 2020 [accessed April 20, 2020]. Database [Internet]. Available from: https://www.seade.gov.br.
