# Supplementary material for: Mathematical modeling of the transmission of SARS-CoV-2—Evaluating the impact of isolation in São Paulo State (Brazil) and lockdown in Spain associated with protective measures on the epidemic of CoViD-19
Source: PLoS One. 2021 Jun 15;16(6):e0252271. doi: 10.1371/journal.pone.0252271 (PMC8205178; doi:10.1371/journal.pone.0252271)
Supplement: Attachment — Submitted filename: Referee1.pdf [file pone.0252271.s002.pdf]

Title: Mathematical modeling of the transmission of SARS-CoV-2 - Evaluating the impact of isolation in São Paulo State (Brazil) and lockdown in Spain associated with protective measures on the epidemic of covid-19.

Ms. ID: PONE-D-20-19611

Comments on the comments of referee #1.

Initially, I must state that we have two set of covid-19 data (both in São Paulo State and Spain) at the beginning of the epidemic, and during the first early months. (1) Severe covid-19 cases (in hospitals, they were tested and confirmed), and (2) deaths due to covid-19.

Reviewer 1 stated that the statistical analysis been performed appropriately and rigorously were not done. Indeed, there is not any statistical approach in the submitted manuscript. Also, reviewer 1 answered no to the question "have the authors made all data underlying the findings in their manuscript fully available?" The links (São Paulo State and Spain data) were cited in the references, and from pages 42 to 45 the data from São Paulo State and Spain were extensively described and discussed.

The marks ? and ?? in the citations and equations can be found in the submitted manuscript.

1) It is too long. Could you compress the paper ?
Reply: The paper is long aiming to clearly explain, and also to be better understood, our approach to the estimation of the model parameters. The severe covid-19 data presented distinct trends, which were explained by estimating the model parameters. The first period corresponds to the natural epidemic (without interventions), used to estimate the transmission rates β_y and β_o (and, then, the basic reproduction number R_0 is calculated). After the introduction of isolation (or lockdown), covid-19 cases follow a decreased trend, which is used to estimate the proportion k of the isolated population. Another decrease in the covid-19 cases is observed, which is the third trend, from which we estimated the reduction in the transmission of the SARS-CoV-2 ε (this parameter describes the reduction due to protective individual (face mask, washing hands) and collective (social distancing) measures). Finally, using the number of deaths due to covid-19, we estimate the lethality (additional mortality) rates α_y and α_o. Besides the better estimated parameters, we showed the epidemic curves obtained using values of the parameters departed from the estimated values. See pages 5 to 10, and pages 15 to 19. I think that these pages could be transported to Appendix.

2) The estimated R0 seems to very large. The author(s) should carefully discuss it. At least, I understand that the author(s) did something wrong. I am wondering that it is reasonable to evaluate R0 at steady state. It might affect the severe upper bias in R0. At least, they have to discuss carefully about other estimated R0 in the same data. Or they have to provide the estimation results in other area and compare them. If the estimation result in other area by the author(s) will be consistently higher than the earlier studies, the author(s) methodology should be concluded to be bias in R0. These robustness check should be necessary.
Reply: With respect to SIR model, we wrote (see page 25 in the submitted paper) "On the other hand, if we estimate the basic reproduction number using SIR model with different

infective persons at $t = 0$, we obtain, using data collected from São Paulo State, $R_0 = 3.22$ (for $I(0) = 10$), or $R_0 = 2.66$ (for $I(0) = 25$), or $R_0 = 2.38$ (for $I(0) = 50$), with other initial conditions being $S(0) = 44.6$ million, and $R(0) = 0$." Notice that in the SIR model, the transmission parameter to calculate R_0 must be estimated considering the infectious individuals (I) as the severe covid-19 cases. The reviewer 1 commented (see comment 10 below) that severe covid-19 cases must not transmit!

Our model is not SIR model. In the manuscript, we described all compartments (see page 28 in the submitted manuscript) "For each subpopulation j ($j = y, o$), all persons are divided into nine classes: susceptible S_j, susceptible persons who are isolated Q_j, exposed and incubating E_j, asymptomatic A_j, symptomatic persons in the initial phase of covid-19 (or pre-diseased) D_{1j}, pre-diseased persons caught by test and then isolated Q_{1j}, symptomatic persons with severe covid-19 D_{2j}, mild covid-19 Q_{2j}, and mild covid-19 persons isolating themselves by educational campaign Q_{3j}. However, all young and elder persons in classes A_j, Q_{1j}, Q_{2j}, Q_{3j}, and D_{2j} enter into the same immune class I (this is the 10th class, but common to both subpopulations)." It is important to stress that in our model severe covid-19 cases do not transmit! The compartments transmitting infection enter in the force of infection λ (see page 28 in the submitted manuscript)

$$\lambda = \frac{1}{N}\frac{\varepsilon}{\omega}\left(\beta_{1y}A_y + \beta_{2y}D_{1y} + \beta_{3y}z_yQ_{2y} + \beta_{1o}A_o + \beta_{2o}D_{1o} + \beta_{3o}z_oQ_{2o}\right).$$

Notice that D_{2y} and D_{2o} are not present in this equation. Also, the ratio between asymptomatic and symptomatic seems to be around 20 (all cases and severe covid-19 cases and seems to be around 100).

Hence, our model is not wrong, but the estimation of R_0 using SIR (or SEIR) model (almost all papers used this model, and considered severe covid-19 cases transmitting the infection), or assuming that R_0 is around 2 is probably wrong.

In SIR model, the dynamical system is (assuming constant number of population)

$$\begin{aligned} \tfrac{d}{dt}S &= \mu(S+I+R) - \beta IS - \mu S \\ \tfrac{d}{dt}I &= \beta IS - (\gamma+\mu)\,I \\ \tfrac{e}{dt}R &= \gamma I - \mu R, \end{aligned} \tag{1}$$

which, in the steady state analysis, we can conclude that the trivial equilibrium is stable if $R_0 < 1$, and unstable otherwise, where

$$R_0 = \frac{\beta}{\gamma+\mu}.$$

However, equation (1) can be written at the very beginning of the epidemic ($S = N - 1 \sim N$, $I = 1$ and $R = 0$)

$$\begin{aligned} \tfrac{d}{dt}S &\sim -\beta N \\ \tfrac{d}{dt}I &\sim (\gamma+\mu)\,(R_0-1)\,I \\ \tfrac{e}{dt}R &= \gamma I - \mu R. \end{aligned}$$

Hence, at the beginning of the epidemic, if we estimate the transmission rate β, we can calculate R_0 using the expression obtained from the steady state analysis. We state this clearly (see page 40 in the submitted manuscript) "The basic reproduction number R_0 obtained from mathematical modelings provides two useful information: At the beginning of the epidemic ($t = 0$), R_0 gives the magnitude of control efforts, and when epidemic reaches the steady-state (after many waves of the epidemic, that is, $t \to \infty$), R_0 measures its severity providing the fraction

of susceptible individuals, in general, $s^* = 1/R_0$ [?]. Between these two extremes, the effective reproduction number R_{ef} dictates the course of an epidemic, which follows decaying oscillations around $R_{ef} = 1$ [?]." Following this explanation, we defined the effective reproduction number R_{ef}.

In several parts of the submitted manuscript the question of higher estimation of R_0 is discussed. For instance, in page 6 we wrote "We stress the fact that, if the observed data are fitted without caution about interventions, that is, using all data indistinctly, someone could estimate the basic reproduction number to be $R_0 = 5.09$ or less (notice that near the horizontal axis of Figure 1(b), the observed data do not approach the curve of $R_0 = 5.09$, rather lower R_0).", and in page 25, "In the literature, the usually assumed basic reproduction number R_0 is around 3, see for instance [?] and [?]. However, Li *et al.* [?] explicitly cited that, by using data from January 10 to February 8, 2020, they estimated the effective reproduction number R_{ef}, arguing that the most recent common ancestor could have occurred on November 17, 2019. The time elapsed from November 17, 2019 (the first case) to January 10, 2020 (the first day in the estimation) is 54 days. On January 23, 2020, Wuhan and other cities of Hubei province imposed lockdown. As we pointed out in Figure 1(b), taking into account the entire data, or restricting the interval of data around the implementation of isolation in São Paulo State, the estimated R_{ef} must be lower than 5.09. From Figures 7 and 14, the effective reproduction number R_{ef} for São Paulo State, 54 days after the beginning of the epidemic, is 2.1 (April 20), while for Spain, $R_{ef} = 0.6$ (March 25). In other words, using covid-19 data beginning from April 20 (São Paulo State) or March 25 (Spain), probably the estimated effective reproduction number will be close to those retrieved from Figures 7 and 14. On the other hand, if we estimate the basic reproduction number using SIR model with different infective persons at $t = 0$, we obtain, using data collected from São Paulo State, $R_0 = 3.22$ (for $I(0) = 10$), or $R_0 = 2.66$ (for $I(0) = 25$), or $R_0 = 2.38$ (for $I(0) = 50$), with other initial conditions being $S(0) = 44.6$ million, and $R(0) = 0$. The reliable estimation of R_0 is important because this number determines the magnitude of effort to eradicate infection. For instance, the efforts of vaccination to eradicate an infection must be vaccinating a fraction equal or greater than $1-1/R_0$ of susceptibles [?]. In [?], analyzing vaccination as a control mechanism, if R_0 is reduced by the vaccine to value lower than one, the number of cases decreased following exponential-type decay, as we observed in Figure 14 describing the lockdown in Spain. Hence, instead of a vaccine, let us consider lockdown to control covid-19 transmission. If $R_0 = 3$, we must isolate at least 67% of the population, while for $R_0 = 8$, at least 87% of the population. As we have pointed out, 70% of the population in lockdown does not explain the covid-19 data in Spain, but 90% of the population in lockdown explained better the observed data. Hence, our estimations of R_0 for São Paulo State (9.24) and Spain (8.0) using the first period of covid-19 data represent the natural epidemic. Moreover, the estimation of R_0 based on the first period is in concordance with the definition of the basic reproduction number: Completely susceptible population without constraints (interventions) [?]."

3) We need to confidence interval of the estimated parameter. If CI of R0 include two, it might not be statistically difference among the earlier study.

Reply: We stated the difficulty (mathematically) to estimate model parameters using observed data in only one compartment (see page 46 in the submitted manuscript) "However, this estimation method of parameters is extremely complex, and considering observed data only in one variable of the dynamic system is not appropriate (see [?]). For this reason, we evaluate the sum of squared differences by varying model parameters. This simplified method of parameter

evaluation does not provide uncertainties associated with the parameters." For this reason, we showed epidemic curves obtained using values departed from the estimated values.

4) I understand that the model in this paper was deterministic SIR model. If so, you mention it in Introduction and never mention about individual based model, which is not related this paper.

Reply: As we explained above, our model is not SIR. Also, we explained how our deterministic model could be improved (see pages 41 and 42 in the submitted manuscript) "The model given by the system of equations (**??**), (**??**) and (**??**) is non-autonomous and non-constant deterministic model. To obtain the basic reproduction number, we dropped out the pulses in isolation and release in the model and transformed into fractions to obtain R_0. This deterministic model provides an overall view of the epidemic. However, some particular/specific features of the epidemic can be dealt with. In our model, we can incorporate the mobility especially of infectious individuals, and apply the technique of finding traveling waves. By doing this, the geographic expansion of SARS-CoV-2 by calculating the velocity of the front of the wave can be achieved. For instance, the dengue expansion in São Paulo State [**?**] and West Nile Virus in American Continent [**?**] were evaluated by the method of finding traveling waves. We did not consider uncertainties in our model parameters, which can be introduced considering the stochastic version. For instance, the transmission rate βdt can be written as $\bar{\beta} dt \pm \sigma_{\bar{\beta}} dB(t)$, where $\sigma_{\bar{\beta}}$ is the standard deviation of average $\bar{\beta}$ and B is the Brownian motion. Gray *et al.* [**?**] analyzed the SIS model to determine the standard deviation $\sigma_{\bar{\beta}}$ and performed computational simulations. Instead of continuously varying the number of individuals in each class, our model can be simulated allowing integer variation in the population. For instance, Freitas [**?**] used a continuous-time stochastic version of the SEIRV (V stands for vaccinated subpopulation) model with integer-valued states. The simulations were done applying the Gillespie algorithm, considering that the transitions between compartments occur randomly according to a Markov chain with transition rates equal to the deterministic rates. A more complex stochastic process can also be applied considering all classes in our model as stochastic variables. For instance, the stochastic process was applied to schistosomiasis transmission [**?**]. Computational modelings can be formulated. There are innumerous methods, being the agent-based model (ABM) the most widespread. ABM is derived from cellular automata letting each cell carrying on much information. For instance, Ferreira *et al.* [**?**] applied cellular automata approach to evaluate the suitability of sterile insect technique to control *Aedes aegipty* when breeding sites are homogeneously and heterogeneously distributed. However, in computational models, after obtaining thousands of trajectories, the mean-field is calculated [**?**] as well as in the stochastic simulation. However, in the stochastic process, the average values can be calculated for the stochastic variables and, in some cases, these averaged variables follow the corresponding deterministic model [**?**]."

5) If you adopt deterministic SIR model without CI, you have to explain carefully the reason why?do they adopt it. I think that it is not meaningless the estimation without CI.

Reply: See previous reply.

6) The estimation of R0 should be done using epidemic curve, which indicates number of patient at onset date. the author(s) might use the data of the number of reported patients in each day. If so, the authors should take delay to report from onset into the model.

Reply: The epidemic curve is the number of severe covid-19 cases (D_2 in our model).

However, the estimation must not do considering the daily reported cases (see equation (15, page 34) in the submitted manuscript), but in the accumulated cases (see equation (14, page 34) in the submitted manuscript). The daily cases (and D_2) can be obtained from the estimated accumulated new cases (see Figure 8, page 14, and Figure 15, page. 21). Notice that the daily registered covid-19 cases is the new cases, not the epidemic curve (D_2 is the new cases minus the outflow).

We are estimating the transmission rates based on the dynamical model. For this reason, we use the covid-19 data to estimate the severe covid-19 accumulated cases. If we use the data at the time of the infection, and we estimate using the severe covid-19 cases, then we must consider a delay. This is not the case. However, if reviewer had read the manuscript, the delay appears in the estimation procedure. For instance, in page 5, we wrote "The effects of isolation implemented on March 24 are expected to appear later on the daily registered cases of severe covid-19 (the sum of incubation and pre-diseased infection periods (see Table 2) is 9.8 days). Hence, the data from February 26 to April 3 of severe covid-19 cases portray the natural epidemic, that is, the transmission of infection is occurring without any kind of intervention. This epidemiological scenario fulfills the definition of the basic reproduction number R_0 (entire population is susceptible in the absence of constraints), allowing its estimation.", and in page 8, "On April 13, 20 days after the beginning of isolation, we observe the first point leaving completely the curve. This new trend can not be explained by an increased proportion of isolation (see Figure 18). To take into account this new tendency of data, in [?] we hypothesized that the using of face mask (protection of mouth and nose), protection of eyes, constant hygiene (washing hands with alcohol and gel) and social distancing may decrease the transmission of infection [?] [?]. Differently to isolation, these protective measures reduce the transmission rates as can be seen in equation (??). We consider that on April 4, 9 days before the observed points leaving consistently the estimated curve of the epidemic with isolation, the protective measures were adopted by population, which reduced the transmission rates from β_y and β_o to $\beta_y' = \varepsilon\beta_y$ and $\beta_o' = \varepsilon\beta_o$."

However, from the onset of symptoms to death, we must consider a delay to estimate the additional mortality rates. If not, the estimated number of deaths is extremely higher. In page 10, we wrote "We estimate the additional mortality rates $\alpha_y = \Gamma\alpha_0$ and α_o taking into account confirmed deaths from March 16 (t_1) to May 20 (t_{66}), and using equation (3). We fix the previously estimated transmission rates $\beta_y = 0.78$ and $\beta_o = 0.90$ (both in $days^{-1}$), the proportion in isolated population $k = 0.53$, and the protective factor $\varepsilon = 0.5$, to evaluate α_0. As we pointed out in Materials and Methods, we fix $\Delta = 15$ *days* and let $\Gamma = 0.26$ in São Paulo State (74% of deaths are occurring in elder persons with severe covid-19 [?]). The evaluated additional mortality rates are $\alpha_y = 0.00185$ and $\alpha_o = 0.0071$ (both in $days^{-1}$).", in page 34, "The number of deaths due to the severe covid-19 cases is

$$\Pi = \Pi_y + \Pi_o, \quad \text{where} \quad \begin{cases} \frac{d}{dt}\Pi_y = \alpha_y D_{2y}, & \text{with} \quad \Pi_y(0) = 0 \\ \frac{d}{dt}\Pi_o = \alpha_o D_{2o}, & \text{with} \quad \Pi_o(0) = 0. \end{cases} \tag{2}$$

In the estimation of the additional mortality rates, we must bear in mind that the time at which new cases and deaths were registered does not have direct correspondence, rather they are delayed by Δ days, that is, $\Pi_y(t + \Delta) = \alpha_y D_{2y}(t)$, for instance.", and in pages 47 and 48, "To estimate the mortality rates α_y and α_o, the let $\alpha_y = \Gamma\alpha_o$, where Γ is provided by the ratio of deaths occurring in young and elder subpopulations. We fix the previously estimated

parameters β_y and β_o, k, ε, and ω, and evaluate

$$\sum_{i=1}^{n} \left[\Pi \left(t_i + \Delta \right) - \Pi^{ob} \left(t_i \right) \right]^2 , \tag{3}$$

by varying the parameter α_o. The accumulated deaths due to covid-19 Π is given by equation (2), with $\Pi(0) = 0$. Notice that the time of registration t_i of deaths must be related to the deaths of new cases Δ times ago, that is, $D_2(t_i - \Delta)$. We use $\Delta = 15$ *days* obtained by analyzing the data from São Paulo State."

7) The author(s) assume rubella is airborne infection, but I do not agree with it. Measles or varicella are airborne infection.

Reply: Covid-19 is transmitted also by droplets, as rubella infection. I used in wide sense the airborne infection. In pages 6 and 7 of the submitted manuscript, we wrote "It is accepted that SARS-CoV-2 is an airborne infection [?]. For instance, for the rubella infection, using seroprevalence data [?] and dealing with the SEIR model in the steady-state, the estimation was $R_0 = 6.71$ [?]. That estimation was done before the mass vaccination against rubella infection, which was the reason to assume that the estimation was the basic reproduction number. However, the rubella virus was circulating probably not in a steady-state, then the estimation would not be R_0, but the effective reproduction number R_{ef}, given by equation (**??**), and the actual value of R_0 must be higher than 6.71. Moreover, Caieiras City in São Paulo State had $30,000$ inhabitants with a demographic density of $264/km^2$ in 1990, which indicates that $R_0 = 6.71$ is under-estimation for rubella transmission in São Paulo State. As we pointed out in Materials and Methods, we have only two moments during the epidemic when R_0 can be estimated from data: At the beginning without any kind of constraints (during the natural epidemic), and when the steady-state is reached, which in general occurs after a long time [?]."

8) Is really protection measure with mask or handwashing reduce R ? I think that reduction in going out before lock down seems to be more important proved by the earlier paper.

Reply: Many days after the introduction of isolation (or lockdown), the epidemic curve followed a decreased trend, which can not be explained by isolation (the proportion in isolation increased smoothly, not decreased). Additionally, WHO declared (warned) the use of face mask and social distancing to avoid the transmission. These protective measures resulted in an additional decrease in the transmission of covid-19. See pages 8 and 9, and also, page 16 in the submitted manuscript.

9) Why the author(s) ignore less susceptible in children though the model has birth rate ? I think less susceptible in children is the most important question to answer for control of COVID-19.

Reply: We really "ignored" many features of the SARS-CoV-2 transmission, such as gender and age (we considered only two age groups), comorbidity, diversity in the population (demographic, social, genetic, behavior, economic activities, etc.), and many others. The model was formulated to address the question of estimating R_0 and explaining the lethality of covid-19. For this reason, our mathematical model was formulated based on the natural history of the infection and different fatality in individuals with age under and above 60 years. Of course, we can consider more than two age groups, however, the basic reproduction number should not be obtained analytically. It was not an easy task to obtain the equation (25, page 38) for two age groups. That equation was obtained thanks to a conjecture proposed by myself. Hence,

the model does not deal with susceptibility, rather deals with difference in lethality in two age groups. A different model must be formulated to analyze susceptibility. Nevertheless, currently, the less susceptibility in children is not clear, and demands more observations. Does reviewer 1 really believe that less susceptibility in children may control covid-19? More than lockdown and adoption of protective measures?

Indeed, the model considered natality rate, which affects very little in the first wave of the epidemic (natural epidemic lasts for few months), which is, however, essential to predict the second wave of the epidemic (for covid-19, the second wave comes after around 15 years or more).

10) I do not agree with high lethality in COVID-19 to achieve herd immunity. Because the severe patient do not contact anyone other than hospila staff and thus almost not infectiousness. If the author(s) do not think so, they have to provide careful discussion.

Reply: I'm wondering from which manuscript (or paper) reviewer stated above affirmation. We wrote (see page 26 in the submitted manuscript) "The concept of herd immunity is associated with the protection provided by immunized persons to a specific subpopulation under a higher risk of death caused by a syndrome or comorbidity. For instance, in the rubella infection, mass vaccination was planned to diminish the infection among pregnant women to reduce the number of congenital rubella syndrome [**?**]. The misunderstanding of the concept of herd immunity, and the rapid dissemination of underestimated R_0 around 3, may have led to misconducting public health policies. For instance, the United Kingdom (at the beginning of epidemic) and Sweden adopted the idea of immunization by natural infection, believing that the "herd immunity" would protect susceptible, especially elder subpopulation. However, the concept of herd immunity must not be understood as immunization by the circulating virus with high lethality but by the vaccine, which decreases the effective reproduction number R_{ef}. The jump down in Figures 7 and 14 can be applied to vaccinating k proportion of the susceptible persons. However, when the isolation and protective measures are implemented in a population, the effective reproduction number R_{ef} jumps down, but this reduction in R_{ef} is temporary and lasts whenever the population maintains adherence to lockdown/isolation and protective measures. These non-pharmaceutical interventions can be understood as transitory herd protection, such as the herd immunity by vaccination (when available) protects especially the elder subpopulation under higher risk of infection and death."

Our model considered that severe covid-19 cases are hospitalized and do not transmit to other individuals in the population (elsewhere we stated that hospital care workers must be protected due to high amount of virus released by severe covid-19 patients in hospitals). May I ask to reviewer 1 why he/she believes in the estimation provided by SEIR model (majority of papers), which considers severe covid-19 transmitting the disease?

11) How do you the effect of lock down ? Can SIR model express lock down even it is complete homogenous model ?

Reply: The deterministic model is an averaged behavior of extremely complex interactions among individuals in a population. We pointed out how the model can be improved (see comment 4). The effect of isolation (lockdown) can be assessed by estimating the proportion of individuals being isolated (pages 7 and 16 in the submitted manuscript). The biological phenomena are extremely complex and unpredictable. However, an epidemic occurring in a determined region leave its particular trajectory, which can be assessed by estimating the model parameters. For this reason, we have different estimation of R_0 for different regions. Indeed,

SIR model can not provide reliable estimation of R_0 (remember that severe covid-19 cases are not transmitting, which reviewer 1 seems to agree), but more realistic model can provide better estimation. We must be aware of that the estimation of R_0 depends on the mathematical model (there are other approaches, such as statistical methods).

Our model is not completely homogeneous. The splitting of the entire population into two subpopulations aimed to consider the "heterogeneity" in transmission (β_y and β_o) and fatality (α_y and α_o).
