# Supplementary material for: Mathematical modeling of the transmission of SARS-CoV-2—Evaluating the impact of isolation in São Paulo State (Brazil) and lockdown in Spain associated with protective measures on the epidemic of CoViD-19
Source: PLoS One. 2021 Jun 15;16(6):e0252271. doi: 10.1371/journal.pone.0252271 (PMC8205178; doi:10.1371/journal.pone.0252271)
Supplement: Attachment — Submitted filename: About_table_2.pdf [file pone.0252271.s003.pdf]

Title: Mathematical modeling of the transmission of SARS-CoV-2 – Evaluating the impact of isolation in São Paulo State (Brazil) and lockdown in Spain associated with protective measures on the epidemic of covid-19

Ms. ID: PONE-D-20-19611R1

Table 2 in the previous version was split into two Tables: Table 1 and Table A.1. This was done to avoid confusion (Reviewer 3) about data not available.

1 Table 2 – Previous version

Table 2 in the previous version is numbered 1 here.

Table 1: Summary of the model parameters ($j = y, o$) and values (rates in $days^{-1}$, time in *days* and proportions are dimensionless). Some values are calculated ($^{\#}$), or obtained from the liteature (*), or estimated (**) or not available yet (***). The life expectancy in Spain is 83.4 *years*.

Symbol	Meaning	Value
μ	Natural mortality rate	$1/(78.4 \times 365)^{*}$
ϕ	Birth rate	$1/(78.4 \times 365)^{*}$
φ	Aging rate	$6.7 \times 10^{-6\#}$
$\sigma_y\,(\sigma_o)$	Incubation rate	$1/5.8\,(1/5.8)^{\#}$
$\gamma_y\,(\gamma_o)$	Recovery rate of asymptomatic persons	$1/12\,(1/14)^{*}$
$\gamma_{1y}\,(\gamma_{1o})$	Infection rate of pre-diseased persons	$1/4\,(1/4)^{*}$
$\gamma_{2y}\,(\gamma_{2o})$	Recovery rate of severe covid-19	$1/12\,(1/21)^{*}$
$\gamma_{3y}\,(\gamma_{3o})$	Infection rate of mild covid-19 persons	$1/13\,(1/16)^{*}$
$\alpha_y\,(\alpha_o)$	Additional mortality rate	$0.00185\,(0.0071)^{**}$
$\eta_y\,(\eta_o)$	Testing rate among asymptomatic persons	$0\,(0)^{***}$
$\eta_{1y}\,(\eta_{1o})$	Testing rate among pre-diseased persons	$0\,(0)^{***}$
$\eta_{2y}\,(\eta_{2o})$	Testing rate among mild covid-19 persons	$0\,(0)^{***}$
$\varpi_y\,(\varpi_o)$	Adherence to protection behavior rate	$0.0\,(0.0)^{***}$
$\xi_y\,(\xi_o)$	Loss of protection behavior rate	$0\,(0)^{***}$
$k_y\,(k_o)$	Proportion of isolated susceptible persons	$0.53\,(0.53)^{**}$
$l_{1y}\,(l_{1o})$	Proportion released at first time t_1	$0\,(0)^{***}$
τ^{is}	Time of the introduction of isolation	March 24
$\tau_{1y}(\tau_{1o})$	Time of the first (i-th) releasing	June 15(June 15)*
$\theta_y\,(\theta_o)$	Treatment rate	$0(0)^{***}$
$\beta_{1y}\,(\beta_{1o})$	Transmission rate due to asymptomatic persons	$0.78\,(0.9)^{**}$
$\beta_{2y}\,(\beta_{2o})$	Transmission rate due to pre-diseased persons	$0.78\,(0.9)^{**}$
$\beta_{2y}\,(\beta_{2o})$	Transmission rate due to mild covid-19 persons	$0.78\,(0.9)^{**}$
$z_y\,(z_o)$	Proportion circulating of mild covid-19 persons	$0.5\,(0.2)^{**}$
ψ	Scaling factor of transmission among elder persons	1.15^{**}
$\chi_y\,(\chi_o)$	Proportion of remaining as asymptomatic persons	$0.98\,(0.95)^{***}$
$p_y\,(p_o)$	Proportion of asymptomatic persons	$0.8(0.8)^{\#}$
$m_y\,(m_o)$	Proportion of mild (non-hospitalized) covid-19	$0.92\,(0.75)^{\#}$

2 Tables 1 and A.1 – Revised version

Table 1 in the revised version is numbered 2 here.

Table 2: Summary of the reduced model parameters ($j = y, o$) and values (rates in $days^{-1}$, time in $days$ and proportions are dimensionless). Some values are calculated ($^{\#}$) or assumed ($^{\$}$) or obtained from the liteature (*) or estimated (**). The values ($^{\&}$) correspond to São Paulo State. For Spain, $\phi = \mu = 1/(83.4 \times 365)$ $days^{-1}$, $\varphi = 1.14 \times 10^{-5}$ $days^{-1}$, and τ^{is} is March 16.

Symbol	Meaning	Value
μ	Natural mortality rate	$1/(78.4 \times 365)^{*\&}$
ϕ	Birth rate	$1/(78.4 \times 365)^{*\&}$
φ	Aging rate	$6.7 \times 10^{-6\#\&}$
$\sigma_y\,(\sigma_o)$	Incubation rate	$1/5.8\,(1/5.8)^{\#}$
$\gamma_y\,(\gamma_o)$	Recovery rate of asymptomatic persons	$1/12\,(1/14)^{*}$
$\gamma_{1y}\,(\gamma_{1o})$	Infection rate of pre-diseased persons	$1/4\,(1/4)^{*}$
$\gamma_{2y}\,(\gamma_{2o})$	Recovery rate of severe CoViD-19	$1/12\,(1/21)^{*}$
$\gamma_{3y}\,(\gamma_{3o})$	Infection rate of mild CoViD-19 persons	$1/13\,(1/16)^{*}$
τ^{is}	Time of the introduction of isolation	March $24^{*\&}$
$z_y\,(z_o)$	Proportion of transmission by mild CoViD-19 persons	$0.5\,(0.2)^{\$}$
$\chi_y\,(\chi_o)$	Proportion of remaining as asymptomatic persons	$0.98\,(0.95)^{\$}$
$p_y\,(p_o)$	Proportion of asymptomatic persons	$0.8(0.8)^{\#}$
$m_y\,(m_o)$	Proportion of mild (non-hospitalized) CoViD-19	$0.92\,(0.75)^{\#}$
ε	Protection factor	**
ω	Reduction factor	**
$k_y\,(k_o)$	Proportion in isolation	**
$\alpha_y\,(\alpha_o)$	Additional mortality rate	**
$\beta_{1y}(\beta_{1o})$	Transmission rate due to asymptomatic persons	**
$\beta_{2y}(\beta_{2o})$	Transmission rate due to pre-diseased persons	**
$\beta_{3y}(\beta_{3o})$	Transmission rate due to mild CoViD-19 persons	**

Table A.1 in the revised version is numbered 3 here.

Table 3: Summary of the general model parameters ($j = y, o$).

Symbol	Meaning
$\eta_y \, (\eta_o)$	Testing rate among asymptomatic persons
$\eta_{1y} \, (\eta_{1o})$	Testing rate among pre-diseased persons
$\eta_{2y} \, (\eta_{2o})$	Testing rate among mild CoViD-19 persons
$\varpi_y \, (\varpi_o)$	Adherence to protection behavior rate
$\xi_y \, (\xi_o)$	Loss of protection behavior rate
$l_{1y} \, (l_{1o})$	Proportion released at first time t_1
$\tau_{1y}(\tau_{1o})$	Time of the first (i-th) releasing
$\theta_y \, (\theta_o)$	Treatment rate
ψ	Scaling factor of transmission among elder persons
