# Supplementary material for: Mathematical modeling of the transmission of SARS-CoV-2—Evaluating the impact of isolation in São Paulo State (Brazil) and lockdown in Spain associated with protective measures on the epidemic of CoViD-19
Source: PLoS One. 2021 Jun 15;16(6):e0252271. doi: 10.1371/journal.pone.0252271 (PMC8205178; doi:10.1371/journal.pone.0252271)
Supplement: Attachment — Submitted filename: Report on the manuscript PONE-D-20-19611_R2.pdf [file pone.0252271.s004.pdf]

Jan 28, 2021

Report on the revised version of the manuscript titled “Mathematical modeling of the transmission of SARS-CoV-2 - Evaluating the impact of isolation in São Paulo State (Brazil) and lockdown in Spain associated with protective measures on the epidemic of covid-19” (Manuscript no. **PONE-D-20-19611R2**)

The authors tried to address the issues raised by the reviewers. However, to my understanding, not all issues are addressed directly and adequately. During the sensitivity analysis, they have assumed the covariances to be zero without explaining the rationale behind it. If the concept of considering covariances occurs, it indicates that the parameters are treated as random but in the entire analysis (mathematical and numerical) all parameters are treated as constants. Moreover the reason of considering one state in a South American country and one country in Europe is not clear. One could have considered many such pairs. There are also some unambiguity in parameter estimation especially R0. Moreover, although there is a sense of heterogeneity on the model, the authors did not explicitly (and mathematically) use this idea in the model evaluation and analysis.

Based on my understanding, I recommend rejecting the paper.
