# Supplementary material for: Mathematical modeling of the transmission of SARS-CoV-2—Evaluating the impact of isolation in São Paulo State (Brazil) and lockdown in Spain associated with protective measures on the epidemic of CoViD-19
Source: PLoS One. 2021 Jun 15;16(6):e0252271. doi: 10.1371/journal.pone.0252271 (PMC8205178; doi:10.1371/journal.pone.0252271)
Supplement: Attachment — Submitted filename: ResponsetoReviewer3.pdf [file pone.0252271.s005.pdf]

Title: Mathematical modeling of the transmission of SARS-CoV-2 – Evaluating the impact of isolation in São Paulo State (Brazil) and lockdown in Spain associated with protective measures on the epidemic of covid-19

Ms. ID: PONE-D-20-19611R1

Please find attached responses to Referee #3.

Reviewer #3: The authors tried to address the issues raised by the reviewers. However, to my understanding, not all issues are addressed directly and adequately. During the sensitivity analysis, they have assumed the covariances to be zero without explaining the rationale behind it. If the concept of considering covariances occurs, it indicates that the parameters are treated as random but in the entire analysis (mathematical and numerical) all parameters are treated as constants. Moreover the reason of considering one state in a South American country and one country in Europe is not clear. One could have considered many such pairs. There are also some unambiguity in parameter estimation especially R0. Moreover, although there is a sense of heterogeneity on the model, the authors did not explicitly (and mathematically) use this idea in the model evaluation and analysis.

Based on my understanding, I recommend rejecting the paper.

Thanks a lot for your careful revision. Your valuable comments helped to revised the manuscript.

(1) During the sensitivity analysis, they have assumed the covariances to be zero without explaining the rationale behind it. If the concept of considering covariances occurs, it indicates that the parameters are treated as random but in the entire analysis (mathematical and numerical) all parameters are treated as constants.

Reply: We agree. If we consider the random parameters, some model parameters may have independent distributions, others not. We included "**if we assume that the parameters have independent distributions, the covariances are zero**". Additionally, we added a simplified approach to evaluate the sensitivity analysis. We included "**An alternative approach to perform the sensitivity analysis is considering the basic reproduction number R_0 as a function of the variables $u_{ji} \in \Theta_j$, with $j = y, o$ and $i = 1, \cdots, 11$. The first-order approximation of the function R_{0j} with respect to the variable u_{ji} varying in $\Delta u_{ji} > 0$ is given by**

$$\Delta R_{0j}^i = R_{0j}\left(u_{ji} \pm \Delta u_{ji}\right) - R_{0j}\left(u_{ji}\right) \approx \pm \frac{\partial R_{0j}}{\partial u_{ji}} \Delta u_{ji}.$$

Instead of ΔR_{0j}^i, we evaluate the quadratic variation $\left(\Delta R_{0j}^i\right)^2$ for each variable u_{ji}. The sum of the quadratic variations is given by

$$\Delta R_{0j}^2 \approx \sum_{i=1}^{11} \left(\frac{\partial R_{0j}}{\partial u_{ji}} \Delta u_{ji} \right)^2,$$

which is equal to $\varrho_{R_{0j}}^2$ obtained by the previous approach." This simple approach is a deterministic version of random parameters.

(2) Moreover the reason of considering one state in a South American country and one country in Europe is not clear. One could have considered many such pairs.

Reply: We agree. For instance, to evaluate lockdown in Europe, someone could choose Spain (44.6 million) and Italy (60 million), even they have different population sizes. However, **to evaluate two different approaches to control the CoViD-19 epidemic, we considered two regions with similar population sizes – Spain (**47.4 **million, lockdown) and São Paulo State (**44.6 **million, partial quarantine)**. (This sentence was included in the revised text.) Different demography and age-distribution resulted in different estimations for the basic reproduction number R_0 and different fatalities patterns. Summarizing, we choose Spain (country) and São Paulo State (state) with similar population sizes to exemplify non-pharmaceutical interventions called suppression and mitigation. The interventions in Spain and São Paulo State may influence mutant variants' appearance in the SARS-CoV-2 (RNA virus) transmission. Should the drastic reduction in the transmission of SARS-CoV-2 in Spain reduce the mutations, avoiding the appearance of virulent variant?

(3) There are also some unambiguity in parameter estimation especially R0.

Reply: Firstly, to obtain R_0, we used a corresponding system of equations in terms of the fractions of the model variables, which presents steady-state. (The original non-constant population system of equations in terms of the sizes of individuals in each compartment does not approach a steady-state.) This expression was obtained using the next generation method to assess the stability of the trivial equilibrium point. Using model parameters' values obtained from the literature (except β's, k and ε), we estimated the transmission rates β's considering the severe CoViD-19 recorded data restricted to the interval before the effects of quarantine appearing (for São Paulo State, from February 26 to April 3); and R_0 was calculated. The next parameter we estimated was the proportion k in quarantine, using the CoViD-19 recorded data from the introduction of the isolation until the observed data separating from the estimated curve (for São Paulo State, from March 24 to April 12). Since this point, the next parameter ε (protective measures) was estimated (for São Paulo State, from April 4 to May 7). The estimated curve using data until May 7 explained the outcoming severe CoViD-19 epidemic quite well until the end of June when relaxation was initiated.

Moreover, we explained in the text that the non-linear least square estimation method is not appropriate when there are observed data in only one variable of the dynamic system (see page 51).

(4) Moreover, although there is a sense of heterogeneity on the model, the authors did not explicitly (and mathematically) use this idea in the model evaluation and analysis.

Reply: We agree. In the text, we have written that our deterministic model can be modified to a stochastic model incorporating randomness in the model parameters. Also, stochastic simulations can be done using the Gillespie algorithm. We aimed to explain severe CoViD-19 recorded data based on a deterministic model, and these approaches can be studied in future works.
