# Supplementary material for: Mathematical modeling of the transmission of SARS-CoV-2—Evaluating the impact of isolation in São Paulo State (Brazil) and lockdown in Spain associated with protective measures on the epidemic of CoViD-19
Source: PLoS One. 2021 Jun 15;16(6):e0252271. doi: 10.1371/journal.pone.0252271 (PMC8205178; doi:10.1371/journal.pone.0252271)
Supplement: Attachment — Submitted filename: ResponsetoReferee5.pdf [file pone.0252271.s007.pdf]

Title: Mathematical modeling of the transmission of SARS-CoV-2 – Evaluating the impact of isolation in São Paulo State (Brazil) and lockdown in Spain associated with protective measures on the epidemic of covid-19

Ms. ID: PONE-D-20-19611R3

Please find attached responses to Reviewer #5.

Dear Dr. Bamidele Tolulope Odumosu (Reviewer #5),

Thanks a lot for your careful revision.

The manuscript is well written and the mathematical model is appreciable. Authors expression of English language too have improved.

Reply: The model presented in this manuscript was formulated and analyzed in March 2020. We hypothesized that both asymptomatic and pre-symptomatic individuals are transmitting SARS-CoV-2, as well as the air transmission. All these hypotheses were considered to explain the rapid spreading of CoViD-19 worldwide, which were confirmed further.

At the beginning of the pandemic, majority of the models (if not all) used severe CoViD-19 cases to estimate the basic reproduction number R0. However, these individuals are not transmitting, since they are isolated attending hospital care. In our model, severe CoViD-19 cases are not transmitting (they are isolated in hospitals), and SARS-CoV-2 was transmitted by asymptomatic, pre-symptomatic and a proportion of mild CoViD-19 cases.

The model parameters were estimated using available data from February 26 to May 7, 2020 (São Paulo State) and January 31 to May 20, 2020 (Spain). Particularly in São Paulo State, the further observed severe CoViD-19 and deaths matched quite well with the model's predictions while the partial quarantine lasted (end-June 2020).

Sincerely

Hyun Mo Yang
