# Supplementary material for: Mathematical modeling of the transmission of SARS-CoV-2—Evaluating the impact of isolation in São Paulo State (Brazil) and lockdown in Spain associated with protective measures on the epidemic of CoViD-19
Source: PLoS One. 2021 Jun 15;16(6):e0252271. doi: 10.1371/journal.pone.0252271 (PMC8205178; doi:10.1371/journal.pone.0252271)
Supplement: Attachment — Submitted filename: Reviewer 4.docx [file pone.0252271.s006.docx]

**Introduction:**

- **Note 1:**

**Scientific background is good. However, the paragraph which describes the symptoms of COVID-19 in page 2 is not important in this paper, I recommend deleting it. Not sufficient previous researches are mentioned, so I recommend adding a bigger paragraph presenting other related researches findings added to reference 7, 8, 10 you already added, examples of related researches:**

DOI: [10.1017/S0950268820002782](https://www.researchgate.net/deref/http%3A%2F%2Fdx.doi.org%2F10.1017%2FS0950268820002782?_sg%5B0%5D=qfVWFvcJqX1f2yCwjAwMNnlRoitpgQZPzd3wL0XtwHlTM3qF2kTk9XoB3sP20zFfZUQHrxyU79Hckfbd8a0tB7W5Cw.qGjLy7eSK1rg6pkb6PC4UCNVgYXyqQyu7Y588JfXlS172cF_I6HDRr5sgIUEFeyJ7qiVJdGzWKldGZJaDbr0Gw)

DOI: [10.1017/dmp.2020.217](https://www.researchgate.net/deref/http%3A%2F%2Fdx.doi.org%2F10.1017%2Fdmp.2020.217?_sg%5B0%5D=18Av-T5B9g_CH_E5VuLDt8OQuihcivTR64upE-7in-Lz17lqSdP68Yr78DNwXDilKFqo7UZWg-KmCMtbQuHm8BjK4Q.rnEEM1R18WmDrTJCTUkjN8xFh_xziIEOmtcsWBR1YXngSl3G3iVXsItfMmXlsKwUpBbb7v02oIxFozv5POcJSw)

DOI: [10.1186/s12916-020-01872-8](https://bmcmedicine.biomedcentral.com/articles/10.1186/s12916-020-01872-8)

DOI:[10.13140/RG.2.2.35066.11202](https://www.researchgate.net/deref/http%3A%2F%2Fdx.doi.org%2F10.13140%2FRG.2.2.35066.11202?_sg%5B0%5D=aFiDdmRkUxNeJ-tU2DfFL0Kln3JbiuByLu6BiwddHMqjIBuw_ccQ-bE8GDZDF1YpoJo1oNAzFtB6Fwac3tnqbSGY7w.0LzdZABmhHS6adT8RQ3gpH-J4igLPw8uLPS_9tvfDB-W30hfHXf-1P9WD8EwDLaLVB3MivpZ_D9LMvsvCPqXxA)

**Note 2: Also, focus more in the introduction on the knowledge gap which is missing in this research. explaining in the introduction what is missing in the previous researches, and why your paper is needed, what is the originality of your paper compared to the previous researches (A small sentence about each of the previous researches and why they have a deficiency or gap in their methodology compared to your methodology)**

**Note 3: Please add a clear/specific sentence regarding the aim or the objective of this research in the last paragraph of the introduction.**

**Note 4: please delete the sentence (In comparison with the lockdown in Spain…) in page 4, it is better to add your analysis later, only write findings from previous researchers in this section.**

**Methods:**

**Note1: The previous reviewers' comments to minimize the manuscript is good, the authors responded well and moved some parts to the Appendix, which made the manuscript better.**

**Note 2: remove (In appendix A) because you mentioned it later again at the end of the paragraph, which is enough and better**

**Note3: remove the aim from the methods (the aim is added to the introduction) start with what you did only**

**Note 4 :(Please rewrite the paragraph of the methodology and describe briefly what you have done in steps to be more clear for the readers as: First, …. Second, …. Then we did…. till the end). It is not so clear in the current form, it will be hard also for readers to follow up and to understand what you have done clearly.**

**Note 3: Specify the period/ time duration of the data you analysed.**

**Note 4: The authors did good job with specifying subgroups, comparability of assessment methods for each, and adding the sensitivity analysis was added a better value to the paper.**

**Results:**

**The results section is so big (page 8-17), this section needs a lot of improvements, so I recommend the following to minimize its size:**

**Note 1: re-organizing the results section and summarizing the results and the numbers in tables instead, this will make it easier for the reader, and this will minimize the size.**

**Note 2: Remove the comparability, the analysis, and your conclusions which you made for the results at the results section in page 10 and the later pages and add it later to the discussion section (only keep the results in this section).**

**Discussion:**

**Note 1: start with summarizing how the key results were compatible with the study aim.**

**Note 2: Put all the interpretation here, your analysis and compatibilities, I recommend doing this in subsections.**

**Note 3: the paper needs more focus on the comparability with previous studies results. Use more comparabilities with studies like those I recommended in the introduction.**

**Note 4: the strengths of the paper are mentioned. However, add a paragraph about all the limitations at the end of the discussion section after the strengths, collect all the points you mentioned previously in the methods section and other limitations, or biases here.**

**Note 5: add a sentence regarding the generalisability (external validity) for other countries and recommendations for other researchers for future research more clearly.**
